# Comparative Efficacy of Chinese Herbal Injections for Treating Pediatric Bronchopneumonia: A Bayesian Network Meta-Analysis of Randomized Controlled Trials

**DOI:** 10.1155/2020/6127197

**Published:** 2020-05-23

**Authors:** Xiaojiao Duan, Haojia Wang, Jiarui Wu, Yubo Guo, Kaihuan Wang, Xinkui Liu, Zeng Xiantao, Xiaomeng Zhang

**Affiliations:** ^1^Department of Clinical Chinese Pharmacy, School of Chinese Materia Medica, Beijing University of Chinese Medicine, Beijing 100102, China; ^2^Standardization Department, China Association of Chinese Medicine, Beijing 100029, China; ^3^Center for Evidence-Based and Translational Medicine, Zhongnan Hospital of Wuhan University, Wuhan 430071, China

## Abstract

**Introduction:**

Pediatric bronchopneumonia is one of the common respiratory diseases in pediatrics. Chinese herbal injections (CHIs) are widely used to treat pediatric bronchopneumonia. In this study, we examined the efficacy of CHIs in the treatment of pediatric bronchopneumonia using a network meta-analysis (NMA).

**Methods:**

Randomized controlled trials (RCTs) of CHIs combined western medicine (WM) versus WM were searched from electronic databases. WinBUGS 1.4.3 and Stata 13.0 were adopted to compute calculations and prepare graphs, respectively.

**Results:**

168 RCTs with 21917 patients were included. The results revealed that Xixinnao injection (XXN) + WM had the most probability to be the best intervention in the four aspects of rate of clinical efficacy, antipyretic time, cough disappearance time, and lung shadow disappearance time. While as to lung shadow disappearance time, asthma disappearance time, and hospitalization time, Yanhuning injection (YHN) + WM could be the best intervention. The safety of CHIs needs to be further assessed.

**Conclusions:**

Based on this NMA, XXN + WM and YHN + WM were potential optimal therapies in pediatric bronchopneumonia, and their safety should be strictly monitored.

## 1. Introduction

Pediatric bronchopneumonia, one of the common respiratory diseases in pediatrics, refers to an inflammatory reaction that is mainly caused by viral, bacterial, or mycoplasma pneumoniae and other pathogenic microorganisms invading the body through the respiratory tract and then clinically resulting in cough, fever, dyspnea, lung rale, and so on [1–3]. With immature respiratory system, weak constitution, and low resistance, it is the bronchial and peripheral lung tissue of children aged 2–5 years that is most susceptible to be invaded by pathogens [4, 5]. Pediatric bronchopneumonia has the characteristics of rapid onset, rapid progress, and easy recurrence. If not treated properly, it will cause various complications, such as cerebral edema, respiratory failure, and heart failure, which seriously affects the health of children [1, 2, 6]. Antibiotics are often used in clinical treatment for pediatric bronchopneumonia. But in recent years, with the long-term use of antibiotics, the drug resistance makes the treatment effect unsatisfactory, its adverse reactions also increasing the physical pain of the child [7]. Currently, a combination between Chinese herbal injections (CHIs) and western medicine (WM) treatment has already achieved some results in treatment of pediatric bronchopneumonia [8–10], but since various CHIs can be chosen, the one with best efficacy is still to be explored. Therefore, this study used a network meta-analysis (NMA) method to comprehensively analyze the efficacy of different CHIs in the treatment of pediatric bronchopneumonia and to obtain a ranking of their clinical effects and then provide reference of evidence-based medicine for clinical rational CHI use.

## 2. Method

This study was reported in strict accordance with the standard format of the specification reported in the Prisma Extension Statement for Reporting of Systematic Reviews Incorporating Network Meta-analyses of Health Care Interventions [11, 12].

### 2.1. Eligibility Criteria and Exclusion Criteria

The eligibility criteria for this study were based on the PICOS principles [13] given in the Cochrane Handbook, including Patient, Intervention, Outcome, and Study design.

Study type: randomized controlled trials (RCTs) of CHIs combined with WM for the treatment of pediatric bronchopneumonia. The article mentioned that “random” can be included. Patient: patients were diagnosed with bronchopneumonia based on definite diagnostic standards, with the age under 15 years, regardless of gender or race. Intervention CHIs combined with WM versus WM alone, or the comparison between different varieties of CHIs combined with WM was performed. Patients received WM treatments (such as antipyretic, cough relief, asthma relief, and anti-infection) according to the specific situation of the child. There was no limitation on the dosages or treatment courses, but other Chinese medicine treatments (such as Chinese herbal decoction, other Chinese patent medicine, acupuncture, and massage) should not be used. Outcomes: the rates of clinical efficacy (RCE), clinical symptom disappearance time (antipyretic time, cough disappearance time, lung rale disappearance time, lung shadow disappearance time, and asthma disappearance time), hospitalization time, and adverse drug reactions/adverse drug events (ADRs/ADEs). The RCE = (the total number of patients − the number of patients ineffective)/the total number of patients ∗ 100% [14–16]. The efficacy evaluation criteria were based on the recovery of clinical symptoms and physiological indicators after treatment, wherein if the clinical symptoms and physiological indicators after treatment were changeless or deteriorate, it was judged to be invalid.

A RCT was excluded if it met any of the following criteria:(1) it was repetitive; (2) the age of the child was not mentioned; (3) the course of treatment was not described; (4) the dosage of CHIs was not described; (5) 2 or more CHIs were used in combination.

### 2.2. Search Strategy

A systematic literature search on RCTs, which referred to the treatment of pediatric bronchopneumonia with CHIs, was performed using the following databases from inception to Apr. 12, 2018: PubMed, Cochrane Library, Embase, Chinese Biomedical Literature Database (SinoMed), China National Knowledge Infrastructure Database (CNKI), Chinese Scientific Journals Full-Text Database (VIP), and Wanfang Database. The references of the relevant literature were also checked. The search strategy included three parts: bronchopneumonia, CHIs, and RCT. 132 kinds of CHIs and 36 chemical injections derived from Chinese medicines that are incorporated in national standards by the National Medical Products Administration were included in our preanalysis. After the preanalysis, we included 6 different CHIs in the NMA: Chuanhuning injection (CHN), Reduning injection (RDN), Tanreqing injection (TRQ), Xixinnao injection (XXN), Xiyanping injection (XYP), and Yanhuning injection (YHN). Detailed searching strategies were illustrated.

### 2.3. Literature Selection and Data Extraction

All articles were managed by NoteExpress software (Wuhan University Library, Wuhan, China). After literature duplicate checking, two reviewers (XD and HW) independently screened potential studies according to the inclusion criteria and extracted data from eligible RCTs; any divergences were resolved by discussion or the third reviewer (JW). The titles and abstracts of literature studies are read to exclude literature studies that were obviously not relevant as well as reviews and animals' experiment reports. Then, full text is read in order to sort out the eligible RCTs. A predesigned data extraction form in Microsoft Excel 2016 was used to manage extract data. The main components of the extracted data were as follows: (1) general information, i.e., author names and publication data; (2) patient information, i.e., number of patients in experimental group and control group, gender, median age or age range, interventions, and treatment; (3) outcomes and outcome measurement data of interest to the study; (4) study types and main factors of risk of bias.

### 2.4. Risk of Bias Assessment

Two authors (KW and XL) assessed the risk of bias in eligible studies independently by using the Cochrane Risk of Bias Tool recommended by the Cochrane Handbook 5.1. Items evaluated were as follows: (1) selection bias associated with random sequence generation; (2) selection bias associated with allocation concealment; (3) blinding of participants and personnel (performance bias); (4) blinding of outcome assessment (detection bias); (5) integrity of outcome data (attrition bias); (6) selective reporting (reporting bias); (7) bias from other sources. There were three levels of bias, namely, “low risk,” “high risk,” and “unclear” for each item. “Low risk” means that the implementation method is correct or does not affect the result; “high risk” means that the implementation method is wrong and affects the measurement of the result; “unclear” means that the information is insufficient and cannot be judged. Consensus was attained by discussion or getting a third opinion (YG).

### 2.5. Statistical Analysis

Statistical analysis was performed with WinBUGS 1.4.3 software (MRC Biostatistics Unit, Cambridge, UK) and Stata 13.0 software (Stata Corporation, College Station, TX, USA). The Markov chain Monte Carlo method with random-effect model was performed for Bayesian inference using the WinBUGS software to carry out the network meta-analysis. In WinBUGS software, the number of iterations was set as 200,000, and the first 10,000 was used for burn-in to eliminate the impact of initial value. For dichotomous outcomes, the pooled results were calculated as odds ratios (ORs). For continuous outcomes, mean differences (MDs) were used. Both types of outcomes were presented with their 95% credible intervals (95% CrIs) as well. When 95% CrIs of ORs did not cover one and 95% CrIs of MDs did not contain zero, differences between the groups were considered statistically significant. Network diagrams of different outcomes were drawn by the Stata software to present relationships among selected CHIs. The results of WinBUGS software calculations were employed by Stata software to calculate the surface under the cumulative ranking curve (SUCRA). An intervention resulting in a larger SUCRA was considered to be the more effective treatment. Therefore, SUCRA was used to evaluate the ranking probabilities for each treatment. A comparison-adjusted funnel plot was used to identify publication bias. If the point distributed in the funnel was symmetrical, there was no publication bias. The method of clustering analysis was utilized to comprehensively compare the effect of CHIs on two different outcomes and then picked the best intervention from these two outcomes. The farther away the origin is in the clustering map, the better the effect in these two outcome indicators. In a closed loop, we employed the inconsistency factor (IF) to evaluate inconsistency among the included studies. If the 95% CrIs of the IF values were truncated at zero, it indicated that there was good consistency.

## 3. Results

### 3.1. Literature Retrieval and Screening Result

**Figure 1:**
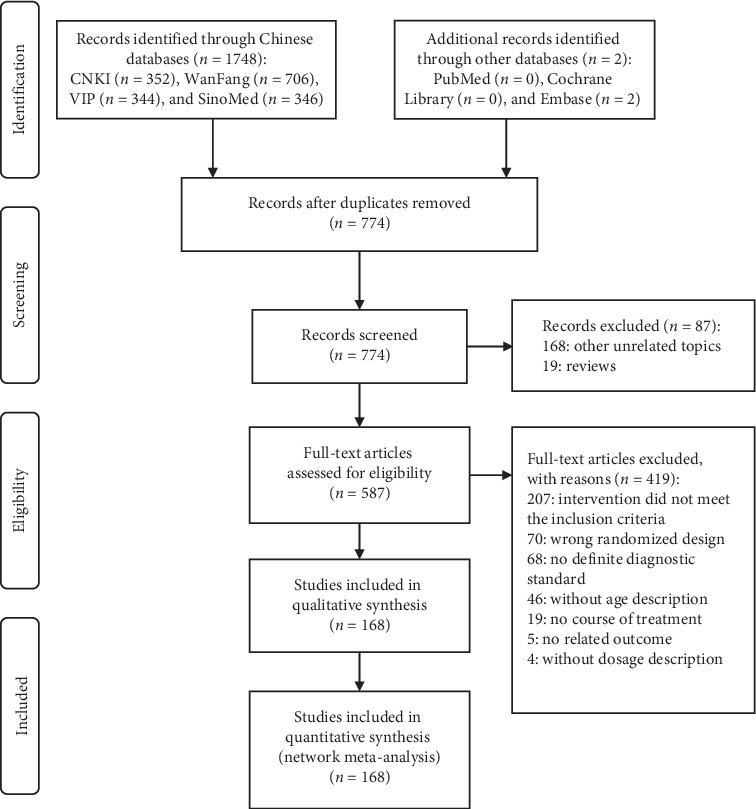
Flow diagram of study search (*n*, number of articles; CNKI, China National Knowledge Infrastructure Database; WanFang, the WanFang Database; VIP, the Chinese Scientific Journals Full-Text Database; SinoMed, the Chinese Biomedical Literature Database).

A total of 1,750 articles were preliminary identified from the aforementioned electronic databases. After filtering layer by layer, 168 RCTs were remained. Further details of the literature screening process are presented in Figure 1. All of the studies were two-arm studies, except one three-arm study (TRQ + WM versus YHN + WM versus XYP + WM). Among them, intervention of 162 studies was CHIs + WM versus WM, including 6 kinds of CHIs, namely, CHN (4 RCTs), RDN (31 RCTs), TRQ (62 RCTs), XXN (12 RCTs), XYP (24 RCTs), and YHN (29 RCTs). The other 5 studies were CHI(a) + WM versus CHI(b) + WM as follows: RDN + WM versus YHN + WM (1 RCT), XYP + WM versus YHN + WM (1 RCT), XYP + WM versus XXN + WM (1 RCT), XYP + WM versus CHN + WM (1 RCT), and TRQ + WM versus YNH + WM (1 RCT). All studies were published in Chinese from 2001 to 2018.

### 3.2. Characteristics of the Included Studies

**Figure 2:**
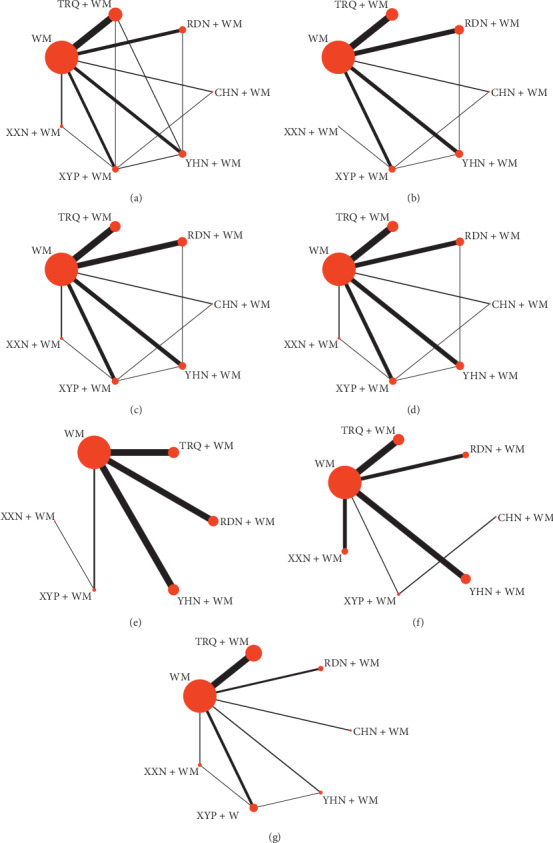
Network graph for different outcomes. (a) RCE; (b) antipyretic time; (c) cough disappearance time; (d) lung rale disappearance time; (e) lung shadow disappearance time; (f) asthma disappearance time; (g) hospitalization time. RDN, Reduning injection; TRQ, Tanreqing injection; XXN, Xixinnao injection; XYP, Xiyanping injection; YHN, Yanhuning injection; CHN, Chuanhuning injection.

The 168 studies included 21917 patients, with sample sizes varying from 15 to 420. Among them, 363 patients accepted the treatment of CHN + WM, 1827 patients accepted the treatment of RDN + WM, 4634 patients accepted the treatment of TRQ + WM, 712 patients accepted the treatment of XXN + WM, 2415 patients accepted the treatment of XYP + WM, and 1802 patients accepted the treatment of YHN + WM, while 10164 patients only accepted WM. Besides four studies that did not report gender composition, there were 11,458 male patients, accounting for 55.55% (11,458/20,627). There were 163 (97.02%) studies reported RCE, and 104 (61.90%), 108 (64.29%), 102 (60.71%), 45 (26.79%), 29 (17.26%), and 45 (26.79%) RCTs reported antipyretic time, cough disappearance time, lung rale disappearance time, lung shadow disappearance time, asthma disappearance time, and hospitalization time. Besides lung shadow disappearance time that only mentioned 5 CHIs, other outcome indicators included all 6 CHIs. The network graphs of the 6 CHIs with different outcomes are depicted in Figure 2. The details of study characteristics are depicted in Table S3.

### 3.3. Risk of Bias Assessment

**Figure 3:**
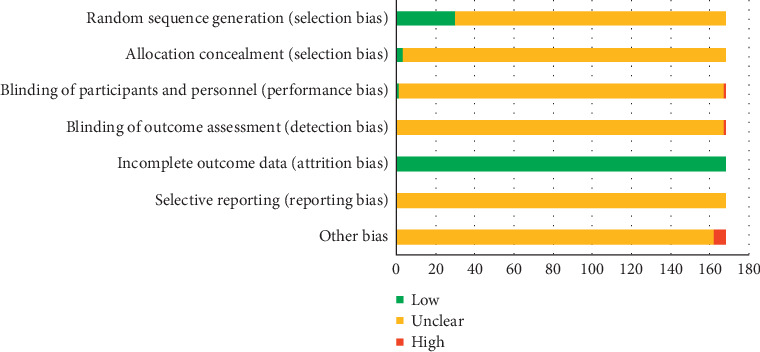
Assessment of risk bias.

In terms of bias assessment, 27 of the 168 studies adequately described their grouping methods as random number table method or lottery to generate the random sequence, so their selection bias was considered to be of low risk due to adequate generation of a randomized sequence; 3 studies were grouped by randomized control principle, and the selective bias caused by random sequence generation and allocation concealment was evaluated as low risk; 1 study used a random single-blind method, and its implementation bias was evaluated as low risk; 1 study used a random unblinded method, and its implementation bias and detection bias were evaluated as high risk; 6 studies did not indicate whether the baseline conditions between two groups were consistent, so the corresponding other biases were evaluated as high risk. Besides, none of the included RCTs had incomplete data, so the attrition bias was appraised as low risk. All the other items were determined as unclear risk when too few details were available to make a decision either way. The details of risk of bias assessment are depicted in Figure 3.

### 3.4. Results of the Network Meta-Analysis

#### 3.4.1. Rate of Clinical Efficacy (RCE)

A total of 163 studies involving 6 CHIs and 7 interventions reported the RCE. The network graph is depicted in Figure 2. The ORs with 95% CrIs of NMA are presented in Table 1. In terms of RCE in treating pediatric bronchopneumonia, CHN + WM, RDN + WM, TRQ + WM, XXN + WM, XYP + WM, and YHN + WM resulted in a significantly better efficacy than WM alone. The differences between the above groups were statistically significant. No statistically significant difference was observed between the other interventions.

**Figure 4:**
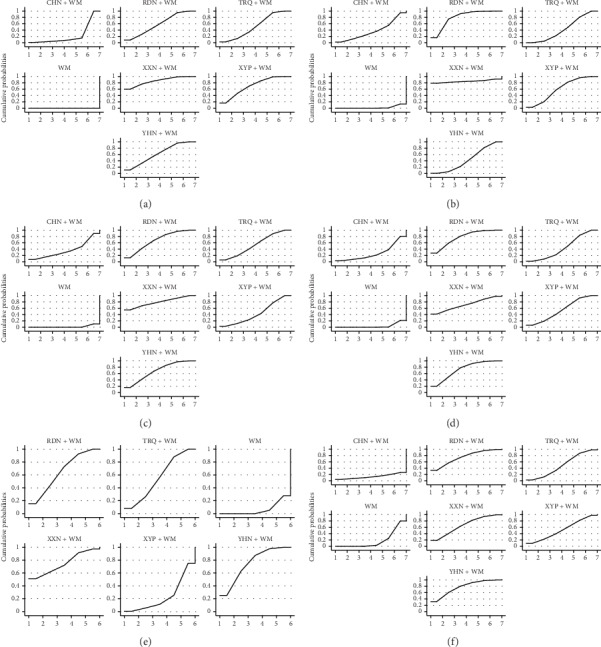
Plots of the surface under the cumulative ranking curves for all treatments. (a) RCE, (b) antipyretic time, (c) cough disappearance time, (d) lung voice disappearance time, (e) lung shadow disappearance time, and (f) asthma disappearance time. RDN, Reduning injection; TRQ, Tanreqing injection; XXN, Xixinnao injection; XYP, Xiyanping injection; YHN, Yanhuning injection; CHN, Chuanhuning injection.

According to results of rank probability based on SUCRA (Figure 4; Table 2), XXN + WM was shown to be the best intervention to improve the RCE, followed by XYP + WM and YHN + WM.

#### 3.4.2. Antipyretic Time

A total of 104 studies involving 6 CHIs and 7 interventions reported the antipyretic time. The network graph is depicted in Figure 2. Outcomes of NMA (Table 1) show that, in terms of reducing antipyretic time, RDN + WM, TRQ + WM, XYP + WM, and YHN + WM resulted in a significantly better benefit than WM alone. The differences between the above groups were statistically significant. No statistically significant difference was observed between the other interventions.

According to results of rank probability based on SUCRA (Figure 4; Table 2), XXN + WM was shown to be the best intervention to reduce antipyretic time, followed by RDN + WM and XYP + WM.

#### 3.4.3. Cough Disappearance Time

A total of 108 studies involving 6 CHIs and 7 interventions reported the cough disappearance time. The network graph is depicted in Figure 2. Outcomes of NMA (Table 1) show that, RDN + WM, TRQ + WM, XXN + WM, XYP + WM, and YHN + WM showed significant benefits on reducing cough disappearance time than WM alone. The differences between the above groups were statistically significant. No statistically significant difference was observed between the other interventions.

According to results of rank probability based on SUCRA (Figure 4; Table 2), XXN + WM was shown to be the best intervention to reduce cough disappearance time, followed by YHN + WM and RDN + WM.

#### 3.4.4. Lung Rale Disappearance Time

A total of 102 studies involving 6 CHIs and 7 interventions reported the lung rale disappearance time. The network graph is depicted in Figure 2. Outcomes of NMA (Table 1) show that, in terms of reducing lung rale disappearance time, RDN + WM, TRQ + WM, XXN + WM, XYP + WM, and YHN + WM were more efficacious than WM alone. The differences between the above groups were statistically significant. No statistically significant difference was observed between the other interventions.

According to results of rank probability based on SUCRA (Figure 4; Table 2), RDN + WM was shown to be the best intervention to reduce lung rale disappearance time, followed by YHN + WM and XXN + WM.

#### 3.4.5. Lung Shadow Disappearance Time

A total of 45 studies involving 5 CHIs without CHN and 6 interventions reported the lung shadow disappearance time. The network graph is depicted in Figure 2. Outcomes of NMA (Table 1) show that, in terms of reducing lung shadow disappearance time, RDN + WM, TRQ + WM, and YHN + WM resulted in a significantly better efficacy than WM alone. The differences between the above groups were statistically significant. No statistically significant difference was observed between the other interventions.

According to results of rank probability based on SUCRA (Figure 4; Table 2), XXN + WM and YHN + WM were shown both to be the best interventions to reduce lung shadow disappearance time, followed by RDN + WM.

#### 3.4.6. Asthma Disappearance Time

A total of 29 studies involving 6 CHIs and 7 interventions reported the asthma disappearance time. The network graph is depicted in Figure 2. Outcomes of NMA (Table 1) show that, in terms of reducing asthma disappearance time, YHN + WM provided significant benefits compared to WM alone. The differences between the above groups were statistically significant. No statistically significant difference was observed between the other interventions.

According to results of rank probability based on SUCRA (Figure 4; Table 2), YHN + WM was shown to be the best intervention to reduce asthma disappearance time, followed by RDN + WM and XXN + WM.

#### 3.4.7. Hospitalization Time

A total of 45 studies involving 6 CHIs and 7 interventions reported the hospitalization time. The network graph is depicted in Figure 2. Outcomes of NMA (Table 1) show that, in terms of reducing hospitalization time, TRQ + WM, XYP + WM, and YHN + WM resulted in a significantly better outcome than WM alone. The differences between the above groups were statistically significant. No statistically significant difference was observed between the other interventions.

According to results of rank probability based on SUCRA (Figure 4; Table 2), YHN + WM was shown to be the best intervention to reduce hospitalization time, followed by XXN + WM and XYP + WM.

### 3.5. Cluster Analysis

**Figure 5:**
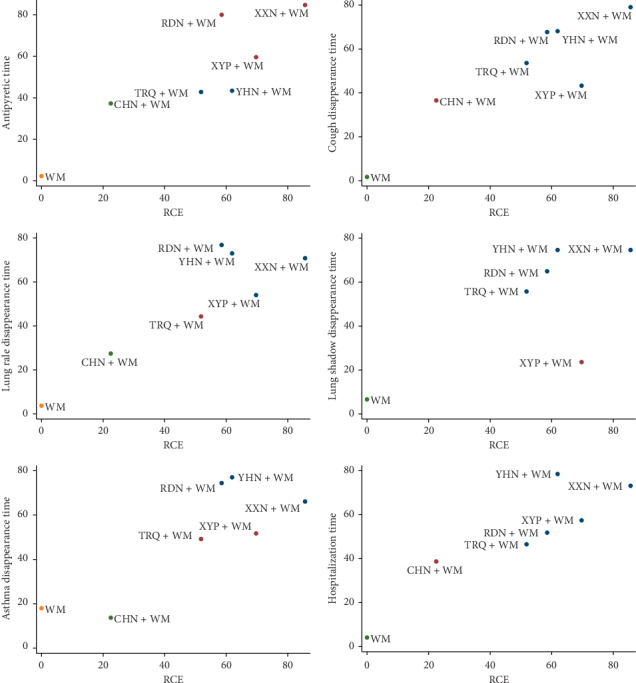
Cluster analysis plots for seven outcomes. Interventions with the same color belonged to the same cluster, and interventions located in the upper right corner indicate optimal therapy for two different outcomes. RDN, Reduning injection; TRQ, Tanreqing injection; XXN, Xixinnao injection; XYP, Xiyanping injection; YHN, Yanhuning injection; CHN, Chuanhuning injection.

The effects of interventions in two different outcomes were comprehensively compared by cluster analysis. Six groups of cluster analysis were performed in this study, including RCE and antipyretic time, RCE and cough disappearance time, RCE and lung rale disappearance time, RCE and lung shadow disappearance time, RCE and asthma disappearance time, and RCE and hospitalization time. The results are presented in Figure 5. Through comprehensive analysis based on cluster analysis, XXN combined with WM was associated with preferable response in any aspect. Its efficacy in the treatment of pediatric bronchopneumonia is worth noting.

### 3.6. Publication Bias

**Figure 6:**
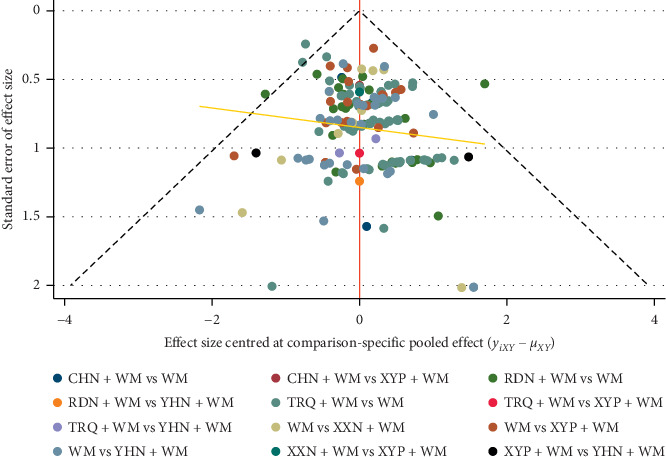
Funnel plot of the clinical effective rate. RDN, Reduning injection; TRQ, Tanreqing injection; XXN, Xixinnao injection; XYP, Xiyanping injection; YHN, Yanhuning injection; CHN, Chuanhuning injection.

A comparison-adjusted funnel plot for the RCE is displayed in Figure 6 to assess whether there was a publication bias. The funnel plot was not completely symmetrical in visual, and the adjusted auxiliary line was not perpendicular to the center line. Thus, the obvious publication bias might exist.

### 3.7. Consistency Test

**Figure 7:**
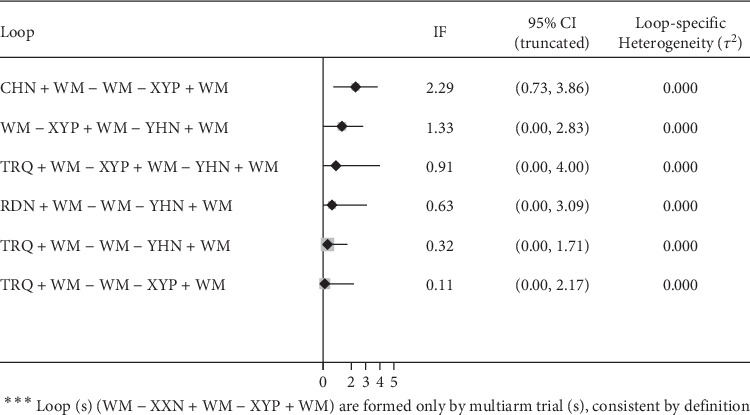
Inconsistency test for the clinical effective rate. RDN, Reduning injection; TRQ, Tanreqing injection; XXN, Xixinnao injection; XYP, Xiyanping injection; YHN, Yanhuning injection; CHN, Chuanhuning injection.

Inconsistency factor and 95% CrIs were calculated with Stata software to evaluate the consistency among closed loops. Zero was not contained in 95% CrI, suggesting a significant inconsistency in the closed loop. Seven loops are shown in Figure 7, and only one loop did not contain zero in its 95% CrI, suggesting a small inconsistency and a reliable result.

### 3.8. Safety

Among 168 included RCTs, 93 (55.36%, 93/168) RCTs addressed ADRs/ADEs during treatment, 12281 (56.03%, 12281/21917) patients were monitored for ADRs/ADEs, 44 studies showed no ADR/ADE, and 49 (52.69%, 49/93) studies clearly indicated the occurrence and number of ADRs/ADEs, while others (44.64%, 75/168) did not monitor ADRs/ADEs during treatment. The details of ADRs/ADEs are represented in Table 3.

## 4. Discussion

Based on the data from 168 enrolled studies with 7 outcome indicators, this study, using network meta-analysis method, systematically evaluated the efficacy of six commonly used CHIs (CHN, RDN, TRQ, XXN, XYP, and YHN) combined with WM in treating pediatric bronchopneumonia. Due to the outcome of NMA, most CHIs combined with WM got better results than using WM only in different outcome indicators, and differences between groups were statistically significant. According to SUCRA results, XXN + WM had the most probability to be the best intervention in the four aspects of RCE, antipyretic time, cough disappearance time, and lung shadow disappearance time. While as to lung shadow disappearance time, asthma disappearance time, and hospitalization time, YHN + WM could be the best intervention. The clustered ranking according to RCE compared with other six outcomes showed XXN + WM was the best intervention. Hence, the efficacy of XXN + WM and YHN + WM in the treatment of pediatric bronchopneumonia deserves our attention, but clinicians should also choose appropriate treatment according to specific conditions of clinical patients.

As for safety, among of the 12 articles that reported in the treatment of pediatric bronchopneumonia with XXN + WM, 6 did not monitor ADR/ADE, and the remaining 6 cases showed no ADRs/ADEs. Therefore, its safety was relatively reliable, but the monitoring was not enough. Among the reported ADRs/ADEs, gastrointestinal reactions accounted for the highest proportion, followed by rash pruritus, which are common ADRs, suggesting that we still should strengthen the monitoring of common ADRs during the course of medication. Judging from ADRs caused by different interventions, XYP + WM and YHN + WM had the highest incidence, suggesting that when using these two interventions for treatment, more attention should be paid.

This study was the first to use a network meta-analysis method to evaluate the efficacy and safety of CHIs for treating pediatric bronchopneumonia. Meanwhile, we ranked the outcomes such as the RCE and clinical symptoms disappearing time in order to provide evidence and recommendations for clinical drug selection. However, this study also had some limitations. First, among 168 studies, there are only 30 studies that used the correct generation method of random sequences, 3 studies used allocation hiding, 1 study used double-blind method, 1 study even used unblinded method, and 6 studies did not describe whether the two sets of baselines were consistent. The quality of the included studies was not high, which reduced the persuasive for the results of the study. Second, limited by application range of CHIs, all studies were done in China and published in Chinese magazines, lacking the data of clinical studies in other languages, which is not conducive to international promotion of research results. Third, most RCTs researched on CHIs + WM versus WM, lacking head-to-head research on comparisons between different CHIs combined with WM. Forth, This study did not limit the types of pathogenic bacteria that cause bronchopneumonia in children, so it was impossible to deeply analyze the efficacy of different CHIs on pediatric bronchopneumonia infected with different strains. No pathogen test result reported in retrieved research was also related. Finally, we did not register our review in PROSPERO.

Based on the above limitations, we made the following suggestions: first, multicenter randomized double-blind trials should be conducted in strict accordance with regulations when developing RCTs. The generation of random sequence, concealment, and blind method implementation were the key points to pay attention to during operation. Second, carry out more clinical studies on the comparative efficacy between different CHIs to compensate for the lack of research in this area. Third, when conducting clinical research on pediatric bronchopneumonia, the types of pathogenic bacteria should be determined first, and appropriate reasonable treatment measures should be selected according to different pathogenic bacteria. Last but not the least, because the condition of bronchopneumonia in children is easy to repeat, follow-up work should be done to monitor the prognosis of children.

## 5. Conclusions

Based on this NMA, CHIs combined with WM provided significant benefits compared to WM alone in treatment of pediatric bronchopneumonia. In addition, among the CHIs, XXN + WM and YHN + WM should deserve our attention. However, due to the limitations of this NMA, our results should be confirmed by more multicenter, larger-sample, and head-to-head RCTs. The safety of CHIs should be strictly monitored.

## Figures and Tables

**Table 1:** Summary of the network meta-analysis for the outcomes (ORs/MDs, 95% CrIs).

	RCE^*∗*^	Antipyretic time	Cough disappearance time	Lung rale disappearance time	Lung shadow disappearance time	Asthma disappearance time	Hospitalization time
*CHN* *+* *WM vs.*								
RDN + WM	1.59 (0.81, 3.06)	0.64 (−0.45, 1.78)	0.62 (−0.94, 2.23)	1.11 (−0.60, 2.82)	—	3.14 (−1.80, 7.83)	0.48 (−2.43, 3.61)	
TRQ + WM	1.53 (0.81, 2.87)	0.15 (−0.91, 1.19)	0.39 (−1.12, 2.09)	0.53 (−1.17, 2.13)	—	2.34 (−2.32, 6.89)	0.37 (−2.10, 2.99)	
XXN + WM	1.97 (0.94, 4.10)	2.61 (−2.26, 7.48)	1.01 (−1.03, 3.06)	1.13 (−1.13, 3.29)	—	2.84 (−1.98, 7.54)	1.46 (−2.53, 5.34)	
XYP + WM	1.70 (0.90, 3.18)	0.35 (−0.64, 1.29)	0.24 (−1.29, 1.94)	0.71 (−0.90, 2.38)	—	2.37 (−1.82, 6.43)	0.68 (−2.03, 3.52)	
YHN + WM	1.62 (0.82, 3.13)	0.14 (−0.93, 1.22)	0.62 (−0.88, 2.32)	1.06 (−0.62, 2.65)	—	3.23 (−1.57, 7.79)	1.32 (−1.39, 3.90)	
WM	**0.33 (0.18, 0.61)**	−0.78 (−1.79, 0.19)	−0.97 (−2.37, 0.57)	−0.67 (−2.22, 0.81)	—	1.49 (−3.07, 5.89)	−1.23 (−3.60, 1.07)	

*RDN* *+* *WM vs.*								
TRQ + WM	0.97 (0.70, 1.35)	−0.51 (−1.07, 0.07)	−0.22 (−1.02, 0.67)	−0.58 (−1.62, 0.46)	−0.25 (−1.82, 1.35)	−0.80 (−2.92, 1.26)	−0.14 (−2.34, 2.03)	
XXN + WM	1.24 (0.76, 2.06)	1.94 (−2.83, 6.69)	0.37 (−1.19, 1.86)	0.01 (−1.86, 1.79)	0.54 (−2.94, 4.16)	−0.31 (−2.68, 2.03)	0.94 (−2.86, 4.57)	
XYP + WM	1.07 (0.73, 1.58)	−0.31 (−1.02, 0.40)	−0.38 (−1.33, 0.60)	−0.40 (−1.46, 0.69)	−1.42 (−3.86, 1.07)	−0.75 (−3.29, 1.73)	0.16 (−2.32, 2.65)	
YHN + WM	1.02 (0.69, 1.51)	−0.50 (−1.12, 0.14)	0.01 (−0.87, 0.85)	−0.09 (−1.01, 0.92)	0.22 (−1.16, 1.68)	0.04 (−2.19, 2.32)	0.83 (−1.54, 3.12)	
WM	**0.21 (0.16, 0.27)**	**−1.44** **(−1.90, −0.98)**	**−1.58** **(−2.18, −0.99)**	**−1.80** **(−2.48, −1.05)**	**−2.10** **(−3.26, −0.93)**	−1.64 (−3.37, 0.11)	−1.74 (−3.75, 0.30)	

*TRQ* *+* *WM vs.*								
XXN + WM	1.29 (0.81, 2.05)	2.45 (−2.36, 7.22)	0.58 (−1.03, 2.19)	0.59 (−1.38, 2.49)	0.80 (−2.66, 4.34)	0.50 (−1.41, 2.40)	1.14 (−2.11, 4.16)	
XYP + WM	1.11 (0.80, 1.54)	0.21 (−0.46, 0.84)	−0.18 (−1.15, 0.79)	0.16 (−0.98, 1.34)	−1.14 (−3.67, 1.20)	0.05 (−2.09, 2.21)	0.31 (−1.28, 1.95)	
YHN + WM	1.06 (0.75, 1.48)	0.00 (−0.58, 0.58)	0.20 (−0.64, 1.10)	0.53 (−0.50, 1.47)	0.49 (−0.89, 1.85)	0.86 (−0.98, 2.69)	0.96 (−0.61, 2.47)	
WM	**0.22 (0.18, 0.26)**	**−0.93 (−1.31, −0.56)**	**−1.37 (−1.96, −0.78)**	**−1.21 (−1.97, −0.46)**	**−1.85 (−2.90, −0.81)**	−0.84 (−1.98, 0.32)	**−1.59 (−2.54, −0.76)**	

*XXN* *+* *WM vs.*								
XYP + WM	0.86 (0.53, 1.38)	−2.24 (−7.02, 2.50)	−0.75 (−2.42, 0.85)	−0.44 (−2.25, 1.56)	−1.96 (−4.55, 0.52)	−0.44 (−2.84, 1.91)	−0.78 (−3.92, 2.46)	
YHN + WM	0.82 (0.49, 1.35)	−2.45 (−7.18, 2.35)	−0.37 (−2.01, 1.21)	−0.10 (−1.94, 1.93)	−0.30 (−3.86, 3.13)	0.34 (−1.73, 2.44)	−0.15 (−3.39, 3.15)	
WM	**0.17 (0.11, 0.26)**	−3.38 (−8.13, 1.40)	**−1.94 (−3.45, −0.55)**	**−1.83 (−3.49, −0.003)**	−2.65 (−6.09, 0.68)	−1.34 (−2.86, 0.18)	−2.72 (−5.70, 0.38)	

*XYP* *+* *WM vs.*								
YHN + WM	0.95 (0.65, 1.40)	−0.19 (−0.84, 0.43)	0.37 (−0.56, 1.35)	0.35 (−0.71, 1.35)	1.640 (−0.69, 4.08)	0.82 (−1.52, 3.11)	0.62 (−1.14, 2.35)	
WM	**0.20 (0.15, 0.26)**	**−1.13 (−1.65, −0.61)**	**−1.19 (−1.97, −0.47)**	**−1.38 (−2.24, −0.61)**	−0.69 (−2.88, 1.60)	−0.88 (−2.69, 0.96)	**−1.92 (−3.32, −0.52)**	

*YHN* *+* *WM vs.*								
WM	**0.21 (0.15, 0.27)**	**−0.94 (−1.38, −0.49)**	**−1.58 (−2.22, −1.00)**	**−1.74 (−2.35, −1.05)**	**−2.34 (−3.22, −1.49)**	**−1.69** **(−3.10, −0.23)**	**−2.55 (−3.75, −1.29)**	

^*∗*^The result is an ORs value; the highlighted result indicates that the difference between the groups is statistically significant. RDN, Reduning injection; TRQ, Tanreqing injection; XXN, Xixinnao injection; XYP, Xiyanping injection; YHN, Yanhuning injection; CHN, Chuanhuning injection.

**Table 2:** Surface under the cumulative ranking probabilities (SUCRA) results of seven outcomes.

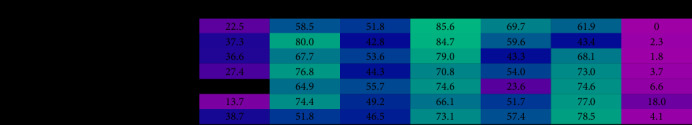

A warm color represents a high SUCRA value, which also suggests a relatively high ranking. RDN, Reduning injection; TRQ, Tanreqing injection; XXN, Xixinnao injection; XYP, Xiyanping injection; YHN, Yanhuning injection; CHN, Chuanhuning injection.

**Table 3:** Details of adverse drug reactions (ADRs)/adverse drug events (ADEs).

	CHN + WM	RDN + WM	TRQ + WM	XYP + WM	YHN + WM	WM	Total number of cases
Gastrointestinal reaction	—	1.09% (15/1379)	0.71% (23/3227)	1.60% (13/814)	3.22% (21/653)	1.61% (91/5651)	163
Rash itch	0.47% (1/212)	0.51% (7/1379)	0.46% (15/3227)	0.49% (4/814)	0.61% (4/653)	0.28% (16/5651)	47
Dizziness	—	0.07% (1/1379)	—	1.84% (15/814)	—	0.28% (16/5651)	32
Antifeeding	—	0.07% (1/1379)	—	—	—	0.11% (6/5651)	7
Increased breathe and heart rate	—	0.07% (1/1379)	—	—	—	—	1
Chill	—	0.07% (1/1379)	—	—	—	—	1
Dilute stool	—	0.15% (2/1379)	0.96% (31/3227)	—	—	—	33
Cough aggravation	—	—	0.06% (2/3227)	—	—	—	2
Inciting	—	—	0.15% (5/3227)	—	—	—	5
Leukopenia	—	—	0.19% (6/3227)	—	—	0.07% (4/5651)	10
Phlebitis	—	—	0.06% (2/3227)	—	—	0.04% (2/5651)	4
Abnormal liver function	—	—	—	—	—	0.02% (1/5651)	1
Not specifically described	—	0.07% (1/1379)	0.03% (1/3227)	—	0.15%(1/653)	0.19% (11/5651)	14
Total	0.47% (1/212)	2.10% (29/1379)	2.63% (85/3227)	3.93%(32/814)	3.98%(26/653)	2.60% (147/5651)	320

## Abbreviations

95% CrIs: 95% credible intervals
ADRs/ADEs: Adverse drug reactions/adverse drug events
CHIs: Chinese herbal injections
CHN: Chuanhuning injection
IF: Inconsistency factor
MDs: Mean differences
NMA: Network meta-analysis
ORs: Odds ratios
RCE: Rate of clinical efficacy
RCTs: Randomized controlled trials
RDN: Reduning injection
SUCRA: The surface under the cumulative ranking curve
TRQ: Tanreqing injection
WM: Western medicine
XXN: Xixinnao injection
XYP: Xiyanping injection
YHN: Yanhuning injection.

## Supplementary Materials

Supplementary Materials Table S3: characteristics of included studies.
