# Supplementary material for: Comparative Efficacy of Chinese Herbal Injections for Treating Pediatric Bronchopneumonia: A Bayesian Network Meta-Analysis of Randomized Controlled Trials
Source: Evid Based Complement Alternat Med. 2020 May 23;2020:6127197. doi: 10.1155/2020/6127197 (PMC7262661; doi:10.1155/2020/6127197)
Supplement: Supplementary Materials — Table S3: characteristics of included studies. [file 6127197.f1.doc]

Table S3. Characteristics of included studies

| Study | Random method | Cases(A/B) | Sex  (M/F) | Age | Interventions  （A vs B） | Dosage of CHIs | Course(d) | Consistent baseline | Outcomes | ADRs/ADEs |
| --- | --- | --- | --- | --- | --- | --- | --- | --- | --- | --- |
| Jiang QL 2007 [14] | Random | 120/120 | 126/114 | A:0.5-13; B:0.5-13 | CHN + WM vs WM | 5-10mg/(kg·d) | 7 | Y | ① | None |
| Xiang YL 2005 [15] | Random | 32/32 | 35/29 | 0-7 | CHN + WM vs WM | 6-10mg/(kg·d) | 7 | Y | ① | None |
| Song J 2004 [16] | Random | 89/72 | 87/74 | 0-12 | CHN + WM vs WM | 15mg/(kg·d) | 7 | Y | ①②③④ | Unclear |
| Wang JH 2001 [17] | Random | 60/49 | 60/49 | 0-12 | CHN + WM vs WM | 15mg/(kg·d) | 10 | Y | ①②③④⑦ | Detail |
| Wei YS 2017 [18] | Random | 46/46 | 56/36 | A:2-12(5.9±1.2); B:3-12(6.1±1.3) | RDN + WM vs WM | 0.6ml/(kg·d) | 5 | Y | ①②③④⑦ | Unclear |
| Tang JH 2017 [19] | Random | 40/40 | 43/37 | A:0.25-8(2.0±0.2); B:0.25-8(1.8±0.3) | RDN + WM vs WM | 0.5-0.8ml/(kg·d) | 7 | Y | ①②③④⑥ | Detail |
| Luo L 2017 [20] | Random | 30/30 | 33/27 | 0.33-8(4.78±1.15) | RDN + WM vs WM | 0.5-0.8ml/(kg·d) | 7 | NR | ①②③⑤ | None |
| Zhou LP 2017 [21] | Random number table | 54/54 | 64/44 | A:0.5-3(1.1±0.4); B:0.4-3(1.2±0.5) | RDN + WM vs WM | 0.5-0.8ml/(kg·d) | 7 | Y | ①②③④⑤ | Unclear |
| Li LY 2017 [22] | Lottery | 40/40 | 42/38 | 1-7(3.56±1.28) | RDN + WM vs WM | 0.3-0.6ml/(kg·d) | 7 | NR | ①②③④⑤ | Unclear |
| Zhang SJ 2017 [23] | Random | 51/51 | 51/51 | A:0.5-11(5.67±4.11); B:0.58-12(6.29±3.71) | RDN + WM vs WM | 0.5ml/(kg·d) | 7 | Y | ①②③④ | Detail |
| Chen DX 2016 [24] | Random | 35/35 | 31/39 | A:0.3-9(5.64±2.07); B:0.5-10(5.38±1.89) | RDN + WM vs WM | 0.5-0.8ml/(kg·d) | 7 | Y | ①②③⑤⑦ | None |
| Lin JD 2016 [25] | Random number table | 100/100 | 111/89 | A:2-9(5.38±0.66); B:2-8(5.29±0.68) | RDN + WM vs WM | 0.3-0.6ml/(kg·d) | 14 | Y | ①②③④⑤ | None |
| Ma SF 2016 [26] | Random | 28/28 | 35/21 | A:3-11(4.3±1.5); B:2-11(4.5±1.6) | RDN + WM vs WM | 0.6ml/(kg·d) | 10 | Y | ①②③④ | Detail |
| Li N 2016 [27] | Random | 25/25 | 25/25 | A:2-12(5.4±2.8); B:2-11(5.7±1.9) | RDN + WM vs WM | 0.6ml/(kg·d) | 7 | Y | ① | None |
| Guo YB 2016 [28] | Random | 80/80 | 72/88 | A:3-8(5.2±1.7); B:3-9(5.7±2.1) | RDN + WM vs WM | 0.5-0.8ml/(kg·d) | 7 | Y | ①②③⑤ | Unclear |
| Zhao J 2015 [29] | Random number table | 100/100 | 121/79 | A:0.5-12; B:0.5-12 | RDN + WM vs WM | 0.5-0.8ml/(kg·d) | 7 | Y | ①②③④⑤⑥ | None |
| Lin SX 2015 [30] | Random | 160/159 | 299/20 | 0.33-10(6.2±0.5) | RDN + WM vs WM | 0.8ml/(kg·d) | 3 | Y | ① | None |
| Shen ZW 2015 [31] | Random | 50/50 | 53/47 | A:0.5-3; B:0.5-3 | RDN + WM vs WM | 0.5-0.8ml/(kg·d) | 5 | Y | ①②③④⑤ | Detail |
| Zhuang XR 2015 [32] | Random | 20/20 | 21/19 | A:0.5-11(5.0±3.6); B:0.67-12(4.8±4.1) | RDN + WM vs WM | 0.6ml/(kg·d) | 5 | Y | ①②③④ | Unclear |
| Liu XF 2014 [33] | Random number table or lottery | 45/45 | 52/38 | A:1.6±0.2; B:1.4±0.3 | RDN + WM vs WM | 0.5-0.8ml/(kg·d) | 7 | Y | ①②③④⑤⑥ | None |
| Chen Y 2014 [34] | Random | 50/50 | 60/40 | A:5.2±4.1; B:5.1±3.4 | RDN + WM vs WM | 0.5-0.7ml/(kg·d) | 7 | Y | ①②③ | Detail |
| Fan Y 2014 [35] | Random | 50/50 | 59/41 | 3-13 | RDN + WM vs WM | 0.6ml/(kg·d) | 5 | Y | ①②③④⑦ | Unclear |
| Dong YP 2014 [36] | Random | 65/65 | 71/59 | A:1.11±0.33; B:1.15±0.34 | RDN + WM vs WM | 0.6ml/(kg·d) | 5-7 | Y | ①②③④ | Detail |
| Huang X 2014 [37] | Random | 50/50 | 56/44 | 1-12(5.63±3.01) | RDN + WM vs WM | 0.8ml/(kg·d) | 3 | Y | ①⑦ | Detail |
| Zhang ZF  2013 [38] | Random | 48/48 | 52/44 | A:2-9(5.42±2.17); B:1-8(5.15±2.43) | RDN + WM vs WM | 0.5ml/(kg·d) | 5 | Y | ①③④⑥ | Unclear |
| Wu YZ 2013 [39] | Random | 49/49 | 53/45 | A:2-12(4.52±1.58); B:2-12(4.46±1.68) | RDN + WM vs WM | 0.5-0.7ml/(kg·d) | 10 | Y | ①②③④⑤ | Detail |
| Yin Y 2013 [40] | Random number table | 66/66 | 69/63 | A:7.2±1.6; B:6.9±1.8 | RDN + WM vs WM | 0.3-0.6ml/(kg·d) | 14 | Y | ①②③⑤⑦ | None |
| Tan FY 2015 [41] | Random | 85/71 | 84/72 | A:1-5; B:1-5 | RDN + WM vs WM | 0.5ml/(kg·d) | 3 | Y | ①②③④ | None |
| Zeng FZ 2012 [42] | Random | 60/60 | 69/51 | A:0.5-12; B:0.5-12 | RDN + WM vs WM | 0.5-1.0ml/(kg·d) | 7 | Y | ① | Detail |
| Zhai M 2011 [43] | Random | 35/35 | NR | 0.42-5(3.5±1.4) | RDN + WM vs WM | 0.5-0.6ml/(kg·d) | 7-14 | NR | ①②③④ | None |
| Qi LJ 2011 [44] | Random number table | 40/40 | 54/26 | 0-8 | RDN + WM vs WM | 0.8ml/(kg·d) | 5 | Y | ①②③④ | Detail |
| Shi YM 2011 [45] | Random number table | 55/55 | 66/44 | A:0.5-12; B:0.5-12 | RDN + WM vs WM | 0.6ml/(kg·d) | 5-7 | Y | ①②③④⑤⑦ | Detail |
| Li GF 2010 [46] | Random number table | 40/41 | 46/35 | A:0.42-3; B:0.42-3 | RDN + WM vs WM | 0.5-0.8ml/(kg·d) | 7 | Y | ①②③④⑤⑥ | Detail |
| Chen YN 2018 [47] | Random | 60/60 | 67/53 | A:4.59-2.07; B:4.64-2.31 | RDN + WM vs WM | 0.3-0.5ml/(kg·d) | 7 | Y | ①②③④ | Unclear |
| Zhang WY 2013 [48] | Random | 120/120 | 121/119 | A:0.5-3(18.5); B:0.5-3(19.3) | RDN + WM vs WM | 0.6-0.8ml/(kg·d) | 7 | Y | ①②③④⑦ | None |
| Zhang C 2017 [49] | Random unblinded | 35/35 | 38/32 | 0-11(5.1±0.6) | TRQ + WM vs WM | 0.3-0.5ml/(kg·d) | 7-10 | Y | ①②③④ | Unclear |
| Dai HY 2017 [50] | Random | 19/19 | 22/16 | 3-8(4.57±1.03) | TRQ + WM vs WM | 0.3-0.5ml/(kg·d) | 7 | Y | ①②③④⑥⑦ | None |
| Wang YC 2017 [51] | Random | 30/30 | 34/26 | A:0.58-6(2.7±0.58); B:0.75-5(2.2±0.75) | TRQ + WM vs WM | 0.3-0.5ml/(kg·d) | 7 | Y | ①⑦ | Unclear |
| Xu JY 2016 [52] | Random | 34/34 | 38/30 | 3-12(6.5) | TRQ + WM vs WM | 0.3-0.5ml/(kg·d) | 7 | Y | ①②③④ | Detail |
| Chen XJ 2016 [53] | Random number table | 50/50 | 62/38 | 0.5-8(2.83±2.53) | TRQ + WM vs WM | 0.3-0.5ml/(kg·d) | 7 | Y | ①②③④⑤ | Unclear |
| Lyu J 2016 [54] | Random | 87/73 | 95/65 | 2-12 | TRQ + WM vs WM | 0.3-0.5ml/(kg·d) | 7 | Y | ①②③④ | None |
| Ji EE 2016 [55] | Lottery | 30/30 | 31/29 | A:0.5-9(4.5±1.2); B:1-8(5.6±0.5) | TRQ + WM vs WM | 0.3-0.5ml/(kg·d) | 7 | Y | ①②③④⑥⑦ | Unclear |
| Cen WJ 2015 [56] | Random | 38/38 | 35/41 | 0.5-8(5.1±2.5) | TRQ + WM vs WM | 0.5ml/(kg·d) | 7 | Y | ①②⑤⑦ | Detail |
| Chen SZ 2015 [57] | Random | 49/49 | 57/41 | 0.17-9(3.45±1.52) | TRQ + WM vs WM | 0.3-0.5ml/(kg·d) | 7-10 | Y | ①②③④⑤⑦ | None |
| Lyu Y 2015 [58] | Random number table | 66/66 | 60/72 | A:1-8(3.84); B:1-8(3.96) | TRQ + WM vs WM | 0.3-0.6ml/(kg·d) | 7 | Y | ①②③④⑥ | Detail |
| Chen XY 2015 [59] | Random number table | 43/43 | 41/45 | A:1-10(5.2±0.7); B:1-11(5.3±0.5) | TRQ + WM vs WM | 300-600mg/(kg·d) | 10 | Y | ①⑦ | None |
| Huang J 2014 [60] | Random | 24/24 | 21/27 | A:0.5-12(5.7±1.8); B:0.5-11(5.1±1.9) | TRQ + WM vs WM | 0.5ml/(kg·d) | 7 | Y | ①②⑤ | Unclear |
| Xiong LH 2014 [61] | Random | 50/50 | 60/40 | 0.75-8 | TRQ + WM vs WM | 0.5-1.0ml/(kg·d) | 7 | Y | ①②③④⑦ | Unclear |
| Guo HM 2014 [62] | Random | 39/39 | 48/30 | 0-12 | TRQ + WM vs WM | 0.3-0.5ml/(kg·d) | 7 | Y | ①②④⑤⑦ | Unclear |
| An XS 2014 [63] | Random comparison principle | 50/50 | 53/47 | 1-11(5.3±0.7) | TRQ + WM vs WM | 50-100mg/(kg·d) | 9 | Y | ① | None |
| Fan HJ 2013 [64] | Random | 50/50 | 52/48 | 0-6 | TRQ + WM vs WM | 0.3-0.5ml/(kg·d) | 7 | Y | ①②③④⑤ | None |
| Wang YF 2013 [65] | Random | 52/52 | 62/42 | A:0.42-11(3.2±1.2); B:0.33-12(3.5±1.6) | TRQ + WM vs WM | 0.3-0.5ml/(kg·d) | 7-10 | Y | ① | Unclear |
| Zheng KX 2013 [66] | Random | 45/45 | 47/43 | A:0.31-10.23(3.16±1.42); B:0.27-10.41(3.29±1.31) | TRQ + WM vs WM | 0.3-0.5ml/(kg·d) | 7 | Y | ①⑦ | Detail |
| Yang SP 2013 [67] | Random | 100/96 | 98/98 | A:2-8(5.6±1.5); B:2-8(5.8±1.6) | TRQ + WM vs WM | 0.5ml/(kg·d) | 5-7 | Y | ①②④ | None |
| Shen ZW 2013 [68] | Random | 54/54 | 68/40 | 2-6 | TRQ + WM vs WM | 0.5-1.0ml/(kg·d) | 7 | Y | ①②③④⑤⑦ | Detail |
| Zou FD 2013 [69] | Random number table | 48/48 | 55/41 | A:0.5-10(3.21±1.45); B:0.5-10(3.35±1.51) | TRQ + WM vs WM | 1.0ml/(kg·d) | 7 | Y | ① | Unclear |
| Jia CX 2013 [70] | Random | 36/36 | 43/29 | 0.5-10 | TRQ + WM vs WM | 0.3-0.5ml/(kg·d) | 7 | Y | ①②③④ | Detail |
| Wang HX 2013 [71] | Random | 74/66 | 76/64 | 0.5-12 | TRQ + WM vs WM | 0.5ml/(kg·d) | 7-10 | Y | ① | None |
| Jiang CX 2013 [72] | Random | 45/45 | 53/37 | A:5.82±2.78; B:5.49±2.58 | TRQ + WM vs WM | 0.3-0.5ml/(kg·d) | 10 | Y | ① | Detail |
| Li XD 2012 [73] | Random | 60/60 | 67/53 | 1-12(2.32±0.78) | TRQ + WM vs WM | 0.3-0.5ml/(kg·d) | 5-14 | Y | ①②③④ | Detail |
| Zhao XZ 2012 [74] | Random | 80/40 | 72/48 | 0.75-8 | TRQ + WM vs WM | 0.3-0.5ml/(kg·d) | 5-7 | Y | ①⑦ | None |
| Han FY 2012 [75] | Random | 25/25 | 25/25 | A:2-7(4.5); B:2.5-7(5) | TRQ + WM vs WM | 0.3-0.5ml/(kg·d) | 7 | Y | ①②③④ | None |
| Wang XW 2012 [76] | Random | 60/60 | 61/59 | A:2.5±1.2; B:2.4±1.3 | TRQ + WM vs WM | 0.5ml/(kg·d) | 5-7 | Y | ① | None |
| Yan SJ 2012 [77] | Random number table | 30/30 | 34/26 | A:0.42-3(1.17±0.35); B:0.5-2.84(1.08±0.32) | TRQ + WM vs WM | 0.3-0.5ml/(kg·d) | 7 | Y | ① | Detail |
| Yang JL 2012 [78] | Random | 80/80 | 75/85 | A:0.75-6(2.5±1.4); B:0.75-5(2.3±1.3) | TRQ + WM vs WM | 0.3-0.5ml/(kg·d) | 7-10 | Y | ①②③④⑤ | Detail |
| Sun H  2011 [79] | Random | 47/47 | 48/46 | 2-13(6.37±1.46) | TRQ + WM vs WM | 20ml | 7 | Y | ①②③④⑤ | Detail |
| Zhong RH 2011 [80] | Random | 65/65 | 58/72 | 0.5-12(5.5) | TRQ + WM vs WM | 0.4-0.6ml/(kg·d) | 5-7 | Y | ①②③④⑤⑥⑦ | None |
| Wang YQ 2011 [81] | Random | 50/50 | 57/43 | A:3.8±3.2; B:4.0±3.3 | TRQ + WM vs WM | 0.3-0.5ml/(kg·d) | 5-7 | Y | ①②③⑤ | Unclear |
| Dang Y  2011 [82] | Random | 50/50 | 49/51 | A:0.50-7; B:0.42-8 | TRQ + WM vs WM | 0.5-0.8ml/(kg·d) | 7 | Y | ① | Unclear |
| Zheng AP 2010 [83] | Random | 58/55 | 64/49 | 0-12 | TRQ + WM vs WM | 0.5-1.0ml/(kg·d) | 7-10 | Y | ① | Unclear |
| 刘同思2010 [84] | Random | 79/41 | 68/52 | 0.67-12 | TRQ + WM vs WM | 10ml | 10 | Y | ①② | Unclear |
| Hu SQ 2010 [85] | Random | 46/46 | 45/47 | A:0.25-10; B:0.42-10 | TRQ + WM vs WM | 0.3-0.5ml/(kg·d) | 7 | Y | ①②③④⑥ | Unclear |
| Goou YS 2010 [86] | Random | 108/88 | 93/103 | 0-12 | TRQ + WM vs WM | 0.5-0.7ml/(kg·d) | 7-10 | Y | ①②③④⑥⑦ | Detail |
| Li R 2010 [87] | Random | 56/44 | 46/54 | 0.5-7 | TRQ + WM vs WM | 0.5-1.0ml/(kg·d) | 5-7 | Y | ① | Unclear |
| Liiu H 2010 [88] | Random | 42/45 | 51/36 | 0-12 | TRQ + WM vs WM | 0.5-1.0ml/(kg·d) | 7-10 | Y | ①②③④ | Detail |
| Zhang FJ 2010 [89] | Random | 132/132 | 142/122 | 1-5 | TRQ + WM vs WM | 0.5ml/(kg·d) | 7 | Y | ①⑦ | Unclear |
| Huang ZZ 2010 [90] | Random number table | 66/66 | 60/72 | A:0.25-8(2.84); B:0.25-8(2.96) | TRQ + WM vs WM | 0.3-0.5ml/(kg·d) | 7 | Y | ①②③④⑥ | Detail |
| Xie YH 2009 [91] | Random | 130/106 | 128/108 | 1-12(7) | TRQ + WM vs WM | 0.5-1.0ml/(kg·d) | 7 | Y | ①⑦ | Detail |
| Zhang ZZ 2009 [92] | Random | 142/146 | 138/150 | 0-12 | TRQ + WM vs WM | 0.5-0.7ml/(kg·d) | 7-10 | Y | ①②③④ | Detail |
| Yang XP 2009 [93] | Random | 42/48 | 50/40 | 0-3 | TRQ + WM vs WM | 0.5-1.0ml/(kg·d) | 7-10 | Y | ①③④⑥⑦ | Detail |
| Jian GJ 2008 [94] | Random | 281/285 | 282/284 | 0-12 | TRQ + WM vs WM | 0.5-1.1ml/(kg·d) | 7-10 | Y | ①②③④⑦ | Detail |
| Jiang YZ 2007 [95] | Random | 76/62 | 66/72 | 0.5-7 | TRQ + WM vs WM | 0.5-1.0ml/(kg·d) | 5-7 | Y | ①⑦ | Unclear |
| Guo JH 2007 [96] | Random | 52/52 | 66/38 | A:3.6±1.2; B:3.7±1.3 | TRQ + WM vs WM | 0.6-0.8ml/(kg·d) | 7 | Y | ①②③④⑤ | Detail |
| Rao XH 2006 [97] | Random | 600/200 | 461/339 | 0-12 | TRQ + WM vs WM | 0.5-1.0ml/(kg·d) | 7-10 | Y | ①②③⑤ | Detail |
| Liu D 2005 [98] | Random | 73/65 | 75/63 | 0-11 | TRQ + WM vs WM | 0.3-0.5ml/(kg·d) | 7 | Y | ①②③④⑦ | None |
| Li JH 2018 [99] | Random number table | 67/66 | 73/60 | A:3-10(7.26±5.02); B:4-9(7.31±4.65) | TRQ + WM vs WM | 0.5-1.0ml/(kg·d) | 7 | Y | ① | Detail |
| Dang QJ 2014 [100] | Random | 35/35 | 39/31 | A:0.5-7(2.4); B:0.42-7(2.5) | TRQ + WM vs WM | 0.5-1.0ml/(kg·d) | 7 | Y | ① | Unclear |
| Hu XD 2014 [101] | Random | 49/49 | 56/42 | 0.67-5(2.6±1.1) | TRQ + WM vs WM | 0.3-0.5ml/(kg·d) | 7 | Y | ①②③④⑤ | None |
| Zhou XQ 2013 [102] | Random double blind | 40/40 | 45/35 | 0.83-10(3.4±3.5) | TRQ + WM vs WM | 0.5ml/(kg·d) | 7 | Y | ①②④ | Unclear |
| Wei JH 2013 [103] | Random | 90/80 | 98/72 | A:0.25-2.59; B:0.25-2.50 | TRQ + WM vs WM | 0.3-0.5ml/(kg·d) | 7 | Y | ①⑦ | Unclear |
| Cai L 2012 [104] | Random | 56/56 | 63/49 | A:0.42-13(6±9); B:0.33-13(6.5±6.5) | TRQ + WM vs WM | 0.3-0.5ml/(kg·d) | 7 | Y | ①②③⑥ | Unclear |
| Tang C 2010 [105] | Random | 30/30 | 36/24 | 0.33-11(5.67±2.04) | TRQ + WM vs WM | 10ml | 7-10 | Y | ①⑦ | None |
| Chen R 2010 [106] | Random comparison principle | 100/100 | 120/80 | 0.33-9(3.1) | TRQ + WM vs WM | 0.5-1.0ml/(kg·d) | 7-10 | Y | ①③ | Unclear |
| Hu Y 2010 [107] | Random number table | 30/30 | 37/23 | 0-3 | TRQ + WM vs WM | 0.5ml/(kg·d) | 7 | Y | ①②③④ | Detail |
| Wang Y 2010 [108] | Random | 219/105 | 174/150 | 0.5-12 | TRQ + WM vs WM | 0.3-0.5ml/(kg·d) | 5-7 | Y | ① | Detail |
| Zhou ZM 2008 [109] | Random | 56/52 | 66/42 | 0.5-12 | TRQ + WM vs WM | 0.5-1.0ml/(kg·d) | 7-10 | Y | ① | Unclear |
| Liu HQ 2006 [110] | Random | 96/72 | 96/72 | 0.5-12 | TRQ + WM vs WM | 0.5-1.0ml/(kg·d) | 7-10 | Y | ① | Unclear |
| Liu YX 2016 [111] | Random | 130/130 | NR | 0.5-7 | XXN+ WM vs WM | 1.0mg/(kg·d) | 7 | Y | ① | Unclear |
| Zeng QL 2015 [112] | Random | 34/34 | 42/26 | A:0-1.8(1.4±0.3); B:0-2(1.5±0.4) | XXN+ WM vs WM | 0.5-1.0mg/(kg·d) | 7 | Y | ① | None |
| Guo YW 2012 [113] | Random | 55/55 | 58/52 | A:0.5-3.0(1.5); B:0.5-3(1.4) | XXN+ WM vs WM | 0.3-0.5mg/(kg·d) | 7-10 | NR | ①⑦ | Unclear |
| Pu HS 2009 [114] | Random | 47/33 | 46/34 | 0-3 | XXN+ WM vs WM | 1.0mg/(kg·d) | 5-7 | Y | ① | None |
| Yang H 2008 [115] | Random | 46/32 | 47/31 | 0-12 | XXN+ WM vs WM | 1.0mg/(kg·d) | 3-5 | Y | ③④⑥ | Unclear |
| Ji SH 2008 [116] | Random | 62/58 | 83/37 | 0.25-7 | XXN+ WM vs WM | 0.5-1.0mg/(kg·d) | 7 | Y | ③④⑥⑦ | Unclear |
| Wang GL 2007 [117] | Random | 70/50 | 69/51 | 0-3 | XXN+ WM vs WM | 0.5mg/(kg·d) | 7 | Y | ①③④⑥⑦ | None |
| Wang QJ 2008 [118] | Random | 50/50 | 56/44 | 0-12 | XXN+ WM vs WM | 1.0mg/(kg·d) | 7 | Y | ①③④⑥ | None |
| Lin B 2008 [119] | Random | 50/50 | 60/40 | 0.17-2.0 | XXN+ WM vs WM | 1.0mg/(kg·d) | 7 | Y | ①③ | None |
| Yang XF 2008 [120] | Random | 35/20 | 32/23 | 0.5-3 | XXN+ WM vs WM | 1.0mg/(kg·d) | 7 | Y | ①⑥ | Unclear |
| Wang YL 2007 [121] | Random | 34/33 | 35/32 | 0.25-2 | XXN+ WM vs WM | 1.0mg/(kg·d) | 7 | Y | ①③④ | None |
| Zhang XJ 2007 [122] | Random | 39/32 | 42/29 | 1-3 | XXN+ WM vs WM | 0.5mg/(kg·d) | 7 | Y | ① | Unclear |
| Wang LD 2017 [123] | Random number table | 50/50 | 53/47 | A:3-13(6.2±3.0); B:3-13(6.8±3.4) | XYP+ WM vs WM | 5mg/(kg·d) | 7 | Y | ① | Detail |
| Wang LF 2017 [124] | Random number table | 15/15 | 16/14 | A:1-5(2.8±1.3); B:1-5(2.7±1.3) | XYP+ WM vs WM | 0.4-0.8ml/(kg·d) | 7 | Y | ②③④ | Detail |
| Pan LW 2015 [125] | Random | 58/58 | 64/52 | A:0.58-5.5(3.09±2.42); B:0.75-6(3.26±1.19) | XYP+ WM vs WM | 5-10mg/(kg·d) | 7 | Y | ①⑦ | Detail |
| Ding SH 2015 [126] | Random | 40/40 | 47/33 | A:0.33-9(3.4±1.0); B:0.5-8(3.2±1.1) | XYP+ WM vs WM | 0.2-0.4ml/(kg·d) | 5-7 | Y | ①②③④⑤ | Unclear |
| Zhao X 2015 [127] | Random comparison principle | 49/40 | 51/38 | 1-6(2.7) | XYP+ WM vs WM | 8mg/(kg·d) | 10-14 | Y | ①②③④⑥⑦ | None |
| Chen YZ 2015 [128] | Random | 27/27 | 31/23 | A:0.43-11(3.1±0.5); B:0.5-11(3.0±0.7) | XYP+ WM vs WM | 5mg/(kg·d) | 7 | Y | ①②③④ | Unclear |
| Ni WR 2014 [129] | Random number table | 45/44 | 49/40 | A:1-5(2.9±1.1); B:1-6(2.7±1.2) | XYP+ WM vs WM | 0.4-0.8ml/(kg·d) | 7 | Y | ①②③④ | Unclear |
| Zhang H 2014 [130] | Random | 70/70 | 68/72 | A:0.5-11(5.13±1.8); B:0.5-12(5.46±1.84) | XYP+ WM vs WM | 0.2-0.4ml/(kg·d) | 7 | Y | ①②③④⑦ | Unclear |
| Luo CL 2014 [131] | Random | 59/59 | 72/46 | A:3.1±1.2; B:3.3±1.2 | XYP+ WM vs WM | 10mg/(kg·d) | 7 | Y | ①②③④⑤ | Unclear |
| Li WW 2014 [132] | Random | 40/40 | 45/35 | 0-7 | XYP+ WM vs WM | 5mg/(kg·d) | 7 | Y | ①②③④⑦ | None |
| Ma FJ 2013 [133] | Random | 50/50 | 59/41 | 0.16-14 | XYP+ WM vs WM | 5-10mg/(kg·d) | 7 | Y | ① | Unclear |
| Chen Y 2013 [134] | Random | 60/60 | 69/51 | A:0.5-11; B:0.6-10 | XYP+ WM vs WM | 0.2-0.4ml/(kg·d) | 7 | Y | ①②③④ | Unclear |
| Zhang JH 2013 [135] | Random | 47/47 | 51/43 | A:0.5-6(3.6±1.5); B:0.6-6.1(3.5±1.6) | XYP+ WM vs WM | 5-8mg/(kg·d) | 14 | Y | ① | Detail |
| Lin YJ 2013 [136] | Random | 66/64 | 69/61 | A:0.3-11(5.39±1.63); B:0.16-10(5.34±1.52) | XYP+ WM vs WM | 1-6ml/d | 7 | Y | ①②③④ | Unclear |
| Shi QJ 2012 [137] | Random | 70/68 | 68/70 | A:0.5-11(5.12±1.79); B:0.5-12(5.47±1.83) | XYP+ WM vs WM | 0.2-0.4ml/(kg·d) | 7 | Y | ①②③④ | Unclear |
| Li ZQ 2012 [138] | Random | 60/60 | 66/54 | A:3.2±1.4; B:3.7±1.3 | XYP+ WM vs WM | 0.2-0.4ml/(kg·d) | 5-7 | Y | ①②③④⑤ | Unclear |
| Luo JC 2011 [139] | Random | 40/40 | 38/42 | 1-7(3.5) | XYP+ WM vs WM | 0.4ml/(kg·d) | 5-7 | Y | ①②③④⑦ | Unclear |
| Liu G 2009 [140] | Random | 98/108 | 109/97 | A:3.0±2.0; B:3.5±2.5 | XYP+ WM vs WM | 0.2-0.4ml/(kg·d) | 7 | Y | ①②③④⑦ | Unclear |
| Min H 2005 [141] | Random | 156/148 | 165/139 | A:0.25-11; B:0.41-10 | XYP+ WM vs WM | 0.2-0.4ml/(kg·d) | 7 | Y | ①②③④ | None |
| Lyu J 2005 [142] | Random | 40/40 | 43/37 | 0-12 | XYP+ WM vs WM | 0.2-0.4ml/(kg·d) | 7 | Y | ① | Detail |
| Xu D 2018 [143] | Random number table | 99/99 | 102/96 | 1-14 (8.3±1.6) | XYP+ WM vs WM | 10mg/(kg·d) | 7 | Y | ②③④ | None |
| Li XY 2017 [144] | Random | 420/420 | NR | A:3-12(8.12±1.01); B:3-12(7.78±1.13) | XYP+ WM vs WM | 200mg/d | 7 | Y | ① | Unclear |
| Hu CW 2016 [145] | Random | 200/189 | 239/150 | A:0.58-7(3.0±0.5); B:0.5-8(3.2±0.7) | XYP+ WM vs WM | 5-10mg/(kg·d) | 7 | Y | ① | Detail |
| Zhu CL 2016 [146] | Random | 320/320 | 300/340 | A:3.65; B:3.7 | XYP+ WM vs WM | 5-10mg/(kg·d) | 7 | Y | ③④⑦ | Unclear |
| Tian J 2018 [147] | Random number table | 40/40 | 43/37 | A:1-14(2.83±0.70); B:1-14(8.31±2.62) | YHN+ WM vs WM | 5-10mg/(kg·d) | 7 | Y | ①②③④⑥⑦ | Detail |
| Long L 2017 [148] | Random | 60/60 | NR | 0.25-5 | YHN+ WM vs WM | 5-8mg/(kg·d) | 7 | Y | ① | Unclear |
| Xu YR 2016 [149] | Random number table | 51/51 | 55/47 | 0.42-３(1.65±1.04) | YHN+ WM vs WM | 10mg/(kg·d) | 7 | Y | ①②③④⑤ | Detail |
| Xiao ZQ 2015 [150] | Random number table | 40/46 | 48/38 | A:4.82±4.26; B:4.16±4.23 | YHN+ WM vs WM | 5-10mg/(kg·d) | 7 | Y | ①②③④ | Unclear |
| Zhao L 2014 [151] | Random | 34/34 | 43/25 | 2-11(5.8) | YHN+ WM vs WM | 5-10mg/(kg·d) | 7 | Y | ①②④⑤ | Unclear |
| Liu Y 2014 [152]^]^ | Random | 40/40 | 49/31 | 0.33-1 | YHN+ WM vs WM | 5-10mg/(kg·d) | 7-10 | Y | ①②③④⑤ | Unclear |
| Sun LQ 2014 [153] | Random | 47/47 | 50/44 | A:1-11(5.59±2.06); B:1-12(5.63±2.17) | YHN+ WM vs WM | 8mg/(kg·d) | 7 | Y | ① | Unclear |
| Zhang FM 2013 [154] | Random | 40/40 | 45/35 | 0.17-1.25(0.69) | YHN+ WM vs WM | 10mg/(kg·d) | 7 | Y | ①③④⑥ | Unclear |
| Zhang CY 2012 [155] | Random | 153/150 | 184/119 | 0.92-10(5.6±1.3) | YHN+ WM vs WM | 5mg/(kg·d) | 5 | Y | ①⑦ | Unclear |
| Pang SY 2012 [156] | Random | 178/172 | 181/169 | A:0.25-10(3.5±1.5); B:0.42-10(3.5±1.5) | YHN+ WM vs WM | 8mg/(kg·d) | 7 | Y | ①②③④ | Unclear |
| Wan X 2012 [157] | Random | 25/25 | 34/16 | A:0.67-13(4.88±1.29); B:0.67-12(4.95±1.26) | YHN+ WM vs WM | 5-10mg/(kg·d) | 7 | Y | ①②③④⑤⑥ | Unclear |
| Jiang F 2012 [158] | Random | 60/60 | 89/31 | 3-8(5.8) | YHN+ WM vs WM | 10mg/(kg·d) | 7 | Y | ①⑦ | Detail |
| Zhang XY 2011 [159] | Random | 40/40 | 45/35 | 0.67-12 | YHN+ WM vs WM | 6mg/(kg·d) | 7 | NR | ①②③④⑤ | Unclear |
| Yu XL 2011 [160] | Random | 58/58 | 68/48 | A:1-9(3.7±1.3); B:1-10(3.8±1.2) | YHN+ WM vs WM | 5-10mg/(kg·d) | 7 | Y | ①②③④⑤ | None |
| Wang WJ 2011 [161] | Random | 40/41 | 36/45 | 0.67-5(2) | YHN+ WM vs WM | 5-10mg/(kg·d) | 7 | Y | ①②③④⑥ | None |
| Pang Y 2011 [162] | Random | 60/60 | 66/54 | A:0-8(3.0±0.8); B:0-9(4.6±1.2) | YHN+ WM vs WM | 5-10mg/(kg·d) | 5-7 | Y | ① | Unclear |
| Feng YH 2010 [163] | Random | 48/47 | 49/46 | 1-12 | YHN+ WM vs WM | 5-10mg/(kg·d) | 5 | Y | ① | Unclear |
| Huang YL 2010 [164] | Random | 56/56 | 67/45 | 0.5-6 | YHN+ WM vs WM | 5-10mg/(kg·d) | 7 | Y | ①②③④⑤ | Detail |
| Liu YD 2009 [165] | Random | 58/58 | 67/49 | 0-6 | YHN+ WM vs WM | 5-10mg/(kg·d) | 7 | Y | ① | Unclear |
| Zhou ZH 2008 [166] | Random | 35/35 | 37/33 | A:0.67-10(3.8); B:0.67-10(3.5) | YHN+ WM vs WM | 5-10mg/(kg·d) | 7 | Y | ①②③④ | Unclear |
| Lin YB 2008 [167] | Random | 48/48 | 62/34 | A:1-9(3.7±1.3)； B:1-10(3.8±1.2) | YHN+ WM vs WM | 5-10mg/(kg·d) | 7 | Y | ①②③④⑤ | None |
| Huang BZ 2007 [168] | Random | 68/62 | 82/48 | 0.5-13 | YHN+ WM vs WM | 5-10mg/(kg·d) | 7-14 | Y | ①②③④⑤⑥ | None |
| Xiao QY 2005 [169] | Random | 50/50 | 55/45 | 0-7 | YHN+ WM vs WM | 5-10mg/(kg·d) | 5 | NR | ①②③④⑤⑥ | Unclear |
| Zeng YD 2005 [170] | Random | 39/39 | 42/36 | A:2-13(3.76); B:3-13(3.98) | YHN+ WM vs WM | 10mg/(kg·d) | 5 | Y | ①②③④ | Unclear |
| Zheng FF 2004 [171] | Random | 78/48 | 71/55 | 0.17-13 | YHN+ WM vs WM | 5-10mg/(kg·d) | 7-14 | Y | ①②③④⑤⑥ | Detail |
| Huang JH 2015 [172] | Random number table | 62/62 | 56/68 | A:(4.11±0.90); B:(4.23±1.06) | YHN+ WM vs WM | 5-10mg/(kg·d) | 6 | Y | ①②③④⑤ | Detail |
| Jiang JG 2017 [173] | Random | 31/31 | 34/28 | 0-2(10.5±1.5) | YHN+ WM vs WM | 10mg/(kg·d) | 7 | Y | ①⑦ | Detail |
| Tan M 2013 [174] | Random | 30/30 | 33/27 | A:0.67-10; B:0.5-11 | YHN+ WM vs WM | 6mg/(kg·d) | 7 | Y | ①②③④⑤ | Unclear |
| Hu H 2008 [175] | Random | 40/40 | 53/27 | 0.5-7 | YHN+ WM vs WM | 5-10mg/(kg·d) | 5 | Y | ①②③④⑤⑥ | Unclear |
| Tu Y 2017 [176] | Random number table | 50/50 | 52/48 | 4-10(7.56±0.33) | YHN + WM vs RDN + WM | RDN:10ml/d; YHN:160mg/d | 3 | Y | ①②③④ | Unclear |
| Lei Q 2016 [177] | Random | 50/50 | 43/57 | 2-7(5.1±2.1); 3-8(5.4±2.3) | XYP + WM vs YHN+WM | XYP:10mg/(kg·d); YHN:10mg/(kg·d) | 7 | Y | ①②③④ | Unclear |
| Zhang AH 2015 [178] | Random | 60/60 | 86/34 | 0.33-5 | XXN + WM vs XYP + WM | XYP:0.2-0.4ml/(kg·d); XXN:0.5mg/(kg·d) | 7 | Y | ①②③④⑤⑦ | None |
| Zeng YZ 2010 [179] | Random | 98/62 | 93/67 | 3.5±2.5 | XYP + WM vs CHN + WM | CHN:10mg/(kg·d); XYP:10mg/(kg·d) | 7 | Y | ①②③④⑥⑦ | Unclear |
| Yin ZY 2010 [180] | Random | 59/61 | 71/49 | 1-14 | TRQ + WM vs YHN + WM | TRQ:0.3-0.5ml/(kg·d); YHN:5-8mg/(kg·d); | 7 | Y | ① | None |
| Wang XQ 2010 [181] | Random | 29/32/28 | 48/41 | 6-12 | TRQ + WM vs YHN + WM vs XYP + WM | TRQ:0.5ml/(kg·d); YHN:10mg/(kg·d); XYP:5mg/(kg·d); | 5 | Y | ① | Unclear |

Note: M male, F female, NR not reported, RDN Reduning injection, TRQ Tanreqing injection, XXN Xixinnao injection, XYP Xiyanping injection, YHN Yanhuning injection, CHN Chuanhuning injection, ①: RCE;②: Antipyretic Time, ③: Cough Disappearance Time, ④: Lung Rale Disappearance Time, ⑤: Lung Shadow Disappearance Time, ⑥: Asthma Disappearance Time, ⑦: Hospitalization Time. WM includes antipyretic, antitussive, phlegm, antiasthmatic, antiviral and other treatment methods.

Reference:

[14] Q.L. Jiang. “Analysis of 120 cases of bronchial pneumonia treated with Chuanhuning,” *[China Medical Herald](javascript:void(0))*, vol. 4, no. 28, pp. 45, 2007.

[15] Y.L. Xiang. “Therapeutic effect of Chuanhuning on bronchiolitis,” *Chinese Journal of Misdiagnostics*, vol. 5, no. 6, pp. 1072, 2005.

[16] J. Song. “Observation on 89 cases of pediatric bronchial pneumonia treated with Chuanhuning injection,” *Journal of Practical Medical Techniques*, vol. 11, no. 3, pp. 334, 2004.

[17] J.H. Wang. “Clinical observation of Chuanhuning injection in the treatment of children with bronchial pneumonia,” *Journal of North China Coal Medical College*, vol. 3, no. 4, pp. 463, 2001.

[18] Y.S. Wei. “Therapeutic effect of amoxicillin and clavulanate potassium needle combined with Reduning on children with bronchial pneumonia,” *Journal of North Pharmacy*, vol. 14, no. 9, pp. 90, 2017.

[19] J.H. Tang, R.L. Tang, Z.J. Huang. “Toxic heat ning injection in the treatment of children with bronchopneumonia,” *Acta Medicinae Sinica*, vol. 30, no. 4, pp. 73, 2017.

[20] L. Luo, Y. Zhou, Y.S. Xiao. “Clinical observation of Reduning injection in the treatment of children patients with pediatric bronchopneumonia for 30 cases,” *Chinese Journal of Modern Drug Application*, vol. 11, no. 11, pp. 90, 2017.

[21] L.P. Zhou. “Study on the application effect of Reduning injection in the treatment of bronchial pneumonia in children,” *Guide of China Medicine*, vol. 15, no. 11, pp. 220, 2017.

[22] L.Y. Li. “Effect of ceftriaxone sodium combined with Reduning injection on children with bronchopneumonia,” *Women's Health Research*, no. 5, pp. 75, 2017.

[23] S.J. Zhang. “Therapeutic effect of Reduning injection combined with ceftriaxone sodium on children with bronchial pneumonia,” *China Health Care & Nutrition*, vol. 27, no. 21, pp. 288, 2017.

[24] D.X. Chen. “Clinical effect of Reduning injection on children with bronchopneumonia for 35 cases,” *World Latest Medicine Information*, vol. 16, no. 99, pp. 149, 2016.

[25] J.D. Lin, D.W. Chen. “Effect evaluation of Reduning injection combined with Latamoxef disodium in treatment of children with bronchial pneumonia,” *China Modern Medicine*, vol. 23, no. 28, pp. 105, 2016.

[26] S.F. Ma. “Therapeutic effect of Reduning injection combined with antibiotics on children with bronchopneumonia,” *Journal of Clinical Medical Literature （ElectronicEdition）*, vol. 3, no. 35, pp. 7064, 2016.

[27] N. Li. “Analysis of the efficacy of adjuvant therapy of Reduning injection on children with bronchopneumonia,” *The Medical Forum*, vol. 20, no. 27, pp. 3782, 2016.

[28] Y.B. Guo. “Reduning Injection in the Treatment of Bronchial Pneumonia in Children for 80 Cases,” *[Chinese Medicine Modern Distance Education of China](javascript:void(0))*, vol. 14, no. 5, pp. 49, 2016.

[29] J. Zhao, Y.Y. Zhu. “Clinical observation on the effect of Reduning injection in the treatment of bronchial pneumonia in children,” *Chinese Pediatrics Of Integrated Traditional And Western Medicine*, vol. 7, no. 6, pp. 625, 2015.

[30] S.X. Lin. “Analysis of the therapeutic effect of integrated traditional Chinese and Western medicine on children with bronchial pneumonia,” *Cardiovascular Disease Journal of Integrated Traditional Chinese and Western Medicine (Electronic)*, vol. 3, no. 7, pp. 33, 2015.

[31] Z.W. Shen. “Clinical effect observation of Re Du Ning injection in the treatment of children with bronchial pneumonia,” *Chinese Community Doctors*, vol. 31, no. 6, pp. 73, 2015.

[32] X.R. Zhuang. “Therapeutic effect of Reduning’s adjuvant treatment for children with bronchial pneumonia,” *[Guide of China Medicine](javascript:void(0))*, vol. 13, no. 5, pp. 202, 2015.

[33] X.F. Liu. “Therapeutic effect of Reduning injection on children with bronchial pneumonia,” *[Chinese Journal of Clinical Rational Drug Use](javascript:void(0))*, vol. 7, no. 32, pp. 52, 2014.

[34] Y. Chen, Y.Y. Ding, M.H. Cao. “Therapeutic effect of Reduning injection on children with bronchopneumonia for 100 cases,” *Jilin Medical Journal*, vol. 35, no. 26, pp. 5805, 2014.

[35] Y. Fan, Y.L. Yang, L. Song. “Clinical observation of Re Du Ning injection combined with amoxicillin-clavulanate potassium in the treatment of children bronchial pneumonia,” *[Chinese Community Doctors](javascript:void(0))*, vol. 30, no. 25, pp. 94, 2014.

[36] Y.P. Dong. “Treatment of children with bronchial pneumonia by Reduning injection for 65 cases,” *[Henan Traditional Chinese Medicine](javascript:void(0))*, vol. 34, no. 9, pp. 1846, 2014.

[37] X. Huang, D.F. Zhang. “Analysis on Integrative Traditional Chinese and Western Medicine Treating 50 Cases of Bronchial Pneumonia in Children,” *World Chinese Medicine*, vol. 9, no. 8, pp. 1005, 2014.

[38] Z.F. Zhang, G.P. Dong, L. Du. “Therapeutic effect of azithromycin combined with Reduning on children with bronchial pneumonia,” *[Guide of China Medicine](javascript:void(0))*, vol. 11, no. 36, pp. 448, 2013.

[39] Y.Z. Wu, Y. Zhang. “Therapeutic effect of Reduning injection combined with azithromycin on children with pneumonia and bronchial pneumonia,” *Strait Pharmaceutical Journal*, vol. 25, no. 12, pp. 143, 2013.

[40] Y. Yin. “Clinical observation on Reduning injection in treating 66 cases of children with bronchial pneumonia,” *Clinical Journal of Chinese Medicine*, vol. 5, no. 14, pp. 17, 2013.

[41] F.Y. Tan, P.F. Wei, W. Li, at al. “Clinical observation on 156 cases of child bronchial pneumonia treated with Reduning combination,” *World Health Digest*, vol. 10, no. 30, pp. 39, 2013.

[42] F.Z. Zeng. “Clinical observation on 60 cases of bronchopneumonia in children treated with Reduning injection,” *World Health Digest*, vol. 5, no. 43, pp. 144, 2012.

[43] M. Zhai, X.L. Li, H.Y. Wu. “Clinical observation on 70 cases of bronchial pneumonia treated with Reduning,” *Chinese Community Doctors*, vol. 13, no. 32, pp. 190, 2011.

[44] L.J. Qi, H. Zhang, X.Y. Zhang. “Therapeutic effect of Reduning injection for adjuvant treatment on children with bronchial pneumonia” *[China Practical Medical](javascript:void(0))*, vol. 6, no. 21, pp. 172, 2011.

[45] Y.M. Shi. “Therapeutic effect of Reduning combined with antibiotics on children with bronchopneumonia,” *Strait Pharmaceutical Journal*, vol. 23, no. 5, pp. 166, 2011.

[46] G.F. Li. “Clinical observation of Reduning injection treatment for children with bronchopneumonia,” *Chinese Pediatrics of integrated Traditional and Western Medicine*, vol. 2, no. 5, pp. 450, 2010.

[47] Y.N. Chen, S. Song. “Clinical observation on 60 cases of children with bronchopneumonia treated with Reduning Injection as an adjuvant therapy,” *Journal of Pediatrics of Traditional Chinese Medicine*, vol. 14, no. 4, pp. 39, 2018.

[48] W.Y. Zhang, Z.H. Li. “Curative observation on Reduning Injection for treating 120 cases of infantile bronchopneumonia,” *Modern Medicine & Health*, vol. 29, no. 1, pp. 56, 2013.

[49] C. Zhang. “Clinical analysis of Tanreqing injection in treating children bronchial pneumonia,” *Yiayao Qianyan*, vol. 7, no. 10, pp. 222, 2017.

[50] H.Y. Dai. “Clinical observation of piperacillin sulbactam combined with Tanreqing in treating children with bronchopneumonia,” *Modern Diagnosis & Treatment*, vol. 28, no. 5, pp. 868, 2017.

[51] Y.C. Wang. “Therapeutic effect of Tanreqing injection on children with bronchial pneumonia,” *[Psychological Doctor](javascript:void(0))*, vol. 23, no. 6, pp. 150, 2017.

[52] J.Y. Xu. “Clinical observation on treatment of infantile bronchial pneumonia with Tanreqing injection combined with amoxicillin,” *World Latest Medicine Information*, vol. 16, no. 42, pp. 87, 2016.

[53] X.J. Chen. “Analysis of Curative Effect of Tanreqing Injection Combined with Azithromycin in Treatment of Bronchopneumonia in Children,” *China Foreign Medical Treatment*, vol. 35, no. 14, pp. 157, 2016.

[54] J. Lyu. “Clinical effect observation of Tanreqing injection combined with ambroxol hydrochloride injection in the treatment of children with bronchopneumonia,” *Clinical Medicine*, vol. 36, no. 4, pp. 19, 2016.

[55] E.E. Ji. “Clinical analysis of Tanreqing in treating children bronchial pneumonia,” *[Journal of Huaihai Medicine](javascript:void(0))*, vol. 34, no. 4, pp. 482, 2016.

[56] W.J. Cen. “Therapeutic effect of ceftriaxone sodium combined with Tanreqing injection on children with bronchial pneumonia,” *For All Health*, vol. 9, no. 19, pp. 149, 2015.

[57] S.Z. Chen. “Clinical analysis of Tanreqing assisted treatment of bronchopneumonia in children,” *Jiangxi Medical Journal*, vol. 50, no. 8, pp. 822, 2015.

[58] Y. Lyu. “To explore the effect of Tanreqing injection on children with acute bronchopneumonia,” *For All Health*, vol. 12, no. 3, pp. 613, 2015.

[59] X.Y. Chen. “Observation on Therapeutic Effect of Tanreqing Injection on Bronchopneumonia in Children,” *Second Summit Forum on Exchange of Experiences in Clinical Emergency and Severe Diseases in 2015*, pp. 1-2, 2015.

[60] J. Huang. “Clinical Observation of Ceftriaxone Combined with Tanreqing in Treatment of Bronchial Pneumonia with Children,” *Journal of Hubei University of Chinese Medicine*, vol. 16, no. 3, pp. 27, 2014.

[61] L.H. Xiong, Y.Y. Li. “Therapeutic effect of Tanreqing combined with cefuroxime sodium on children with bronchopneumonia,” *Chinese Journal of Clinical Rational Drug Use*, vol. 7, no. 36, pp. 117, 2014.

[62] H.M. Guo, A.N. Bai. “Therapeutic effect of Tanreqing injection on children with bronchial pneumonia,” *Capital Medicine*, vol. 21, no. 22, pp. 74, 2014.

[63] X.S. An, J. Zhu. “Therapeutic effect of Tanreqing injection on children with bronchial pneumonia,” *China Health Industry*, vol. 11, no. 22, pp. 195, 2014.

[64] H.J. Fan, L. Huang. “Observation of curative effect of Tanreqing injection on children with bronchial pneumonia,” *[Heilongjiang Medicine Journal](javascript:void(0))*, vol. 8, no. 6, pp. 1058, 2013.

[65] Y.F. Wang. “Treatment of 52 cases of infantile bronchial pneumonia with Tanreqing injection,” *China Pharmaceuticals*, vol. 22, no. 6, pp. 118, 2013.

[66] K.X. Zheng. “Observation of efficacy and safety of cefoxitin sodium combined with Tanreqing injection in children with bronchopneum,” *Chinese Journal of Ethnomedicine and Ethnopharmacy*, vol. 22, no. 11, pp. 110, 2013.

[67] S.P. Yang, L. Jiang, Y. Li. “Therapeutic effect of Tanreqing injection as adjuvant therapy on children with acute bronchial pneumonia,” *Chinese Journal of Clinical Rational Drug Use*, vol. 6, no. 5, pp. 58, 2013.

[68] Z.W. Shen. “Clinical observation of Tanreqing injection in treating acute bronchial pneumonia in children,” *[Global Traditional Chinese Medicine](javascript:void(0))*, vol. 6, no. S2, pp. 153, 2013.

[69] F.D. Zou. “Therapeutic effect of Tanreqing injection on children with bronchial pneumonia,” *Jiangxi Medical Journal*, vol. 48, no. 12, pp. 1243, 2013.

[70] C.X. Jia. “Therapeutic effect of Tanreqing injection on children with bronchial pneumonia,” *[Journal of New Chinese Medicine](javascript:void(0))*, vol. 45, no. 7, pp. 99, 2013.

[71] H.X. Wang. “Therapeutic effect of Tanreqing injection on children with bronchial pneumonia,” *Inner Mongol Journal of Traditional Chinese Medicine*, vol. 32, no. 3, pp. 14, 2013.

[72] C.X. Jiang, D.D. Liang, D.M. Yao. “Therapeutic effect of Tanreqing combined with ceftazidime on children with bronchial pneumonia,” *Health Horizon*, vol. 10, no. 1, pp. 71, 2013.

[73] X.D. Li, Y.P. Zhou. “Clinical safety and efficacy evaluation of Tanreqing injection combined with antibiotics in children with bronchopneumonia,” *Guide of China Medicine*, vol. 10, no. 23, pp. 618, 2012.

[74] X.Z. Zhao. “Clinical observation on 80 cases of Children with bronchial pneumonia treated by Tanreqing,” *Jilin Medical Journal*, vol. 33, no. 17, pp. 3658, 2012.

[75] F.Y. Han. “Therapeutic effect of Tanreqing injection on children with bronchial pneumonia,” *Journal of Emergency in Traditional Chinese Medicine*, vol. 21, no. 9, pp. 1529, 2012.

[76] X.W. Wang. “Clinical observation on adjuvant treatment of bronchial pneumonia with Tanreqing injection,” *Journal of Emergency in Traditional Chinese Medicine*, vol. 21, no. 5, pp. 825, 2012.

[77] S.J. Yan, Q.S. Pan, B.W. Zhou, et al. “Clinical Observation on the Ceftriaxone Combined with Tanreqing Injection in Treating Bronchial Pneumonia,” *Journal of Pediatric Pharmacy*, vol. 18, no. 1, pp. 21, 2012.

[78] J.L. Yang. “Clinical observation on treatment of bronchial pneumonia with Tanreqing injection,” *China Health Care & Nutrition*, vol. 22, no. 20, pp. 4627, 2012.

[79] H. Sun, H.M. Sun. “Clinical effect and safety evaluation of Tanreqing injection combined with antibiotics in children with bronchopneumonia,” *Strait Pharmaceutical Journal*, vol. 23, no. 11, pp. 99, 2011.

[80] R.H. Zhong, H.X. Zhang. “Clinical Analysis of 65 Cases of Infantile Bronchial Pneumonia Treated by Integrative Chinese and Western Medicine,” *China Foreign Medical Treatment*, vol. 30, no. 19, pp. 50, 2011.

[81] Y.Q. Wang, G.X. Qu, D.X. Zhou. “Therapeutic effect of Tanreqing on children with bronchial pneumonia,” *Medical Innovation of China*, vol. 8, no. 5, pp. 57, 2011.

[82] Y. Dang, G. Yang. “Therapeutic effect of integrated traditional Chinese and Western medicine on children with bronchopneumonia,” *Journal of Practical Traditional Chinese Medicine*, vol. 27, no. 6, pp. 395, 2011.

[83] A.P. Zheng. “Clinical Observation on 58 Cases of Infantile Bronchial Pneumonia Treated by Tanreqing Injection,” *Chinese Journal of Ethnomedicine and Ethnopharmacy*, vol. 19, no. 13, pp. 170, 2010.

[84] T.E. Liu. “Clinical Observation on 79 Cases of Infantile Bronchial Pneumonia Treated by Tanreqing Injection Combined with Cefoperazone Sodium and Sulbactam Sodium,” *Clinical Journal of Medical Officers*, vol. 38, no. 1, pp. 130, 2010.

[85] S.Q. Hu, F. Su, Z.B. Zhou. “The effect observation of Tanreqing injection in children with bronchial pneumonia,” *Chinese Journal of Clinical Rational Drug Use*, vol. 3, no. 22, pp. 14, 2010.

[86] Y.S. Gou. “Clinical Observation on 108 Cases of Bronchial Pneumonia Treated with Tanreqing Injection,” *China Modern Medicine*, vol. 17, no. 35, pp. 95, 2010.

[87] R. Li, J.M. Wang. “Therapeutic effect of Tanreqing injection on children with bronchial pneumonia,” *Inner Mongol Journal of Traditional Chinese Medicine*, vol. 29, no. 8, pp. 7, 2010.

[88] H. Liu. “Adjuvant Treating 42 Cases of Pediatric Bronchial Pneumonia with Tanreqing Injection,” *Henan Traditional Chinese Medicine*, vol. 30, no. 2, pp. 197, 2010.

[89] F.J. Zhang. “Therapeutic effect of Tanreqing injection on children with bronchial pneumonia,” *World Health Digest*, vol. 7, no. 13, pp. 9, 2010.

[90] Z.Z. Huang. “Clinical observation on the effect of Tanreqing injection in treating children with acute bronchial pneumonia,” *[Chinese Pediatrics of Integrated Traditional and Western Medicine](javascript:void(0))*, vol. 2, no. 2, pp. 173, 2010.

[91] Y.H. Xie. “Clinical Observation on 130 Cases of Acute Bronchial Pneumonia in Children Treated with Tanreqing Injection,” *Yunnan Journal of Traditional Chinese Medicine and Materia Medica*, vol. 30, no. 12, pp. 40, 2009.

[92] Z.Z. Zhang, Y.Q. Li. “Clinical Observation on 142 Cases of Pediatric Bronchial Pneumonia Treated by Tanreqing Injection,” *Chinese Community Doctors*, vol. 11, no. 23, pp. 151, 2009.

[93] X.P. Yang, X.L. Li. “Therapeutic effect of Tanreqing injection on infants with bronchial pneumonia,” *Chinese Journal for Clinicians*, vol. 37, no. 12, pp. 51, 2009.

[94] G.J. Jian, X.H. Sun, C.L. Zhou. “Treatment of 281 Cases of Infantile Bronchial Pneumonia with Tanreqing Injection,” *Modern Journal of Integrated Traditional Chinese and Western Medicine*, vol. 17, no. 3, pp. 375, 2008.

[95] Y.Z. Jiang, M.Z. Zhu. “CLINICAL EFFECT OF TANREQING INJECTION ON CHILDREN WITH BRONCHOPNEUMONIA,” *Acta Academiae Medicinae Qingdao Universitatis*, vol. 43, no. 5, pp. 441, 2007.

[96] J.H. Guo, L.Z. Hou, Z.X. Guo. “Curative Effect Observation on Tanreqing Injection in Treatment of Children's Bronchopneumonia,” *[JOURNAL OF BETHUNE MILITARY MEDICAL COLLEGE](javascript:void(0))*, vol. 5, no. 3, pp. 136, 2007.

[97] X.H. Rao, Q. Tian, H.Y. Chen, et al. “Effects of Tanreqing infection in treatment of acute pneumionia of children,” *[Maternal and Child Health Care of China](javascript:void(0))*, vol. 22, no. 19, pp. 2723, 2007.

[98] D. Liu. “Treatment of 73 cases of children with bronchial pneumonia by combination of antipyretic and antibiotics,” *[MEDICINE INDUSTRY INFORMATION](javascript:void(0))*, vol. 2, no. 23, pp. 86, 2005.

[99] J.H. Li, L. Zhu, X.H. Guo. “Clinical analysis of Tanreqing injection in the treatment of bronchial pneumonia,” *[Journal of Clinical Pulmonary Medicine](javascript:void(0))*, vol. 23, no. 3, pp. 558, 2018.

[100] Q.J. Dang, J.X. Cui. “Therapeutic effect of Tanreqing on non-mycoplasmal bronchopneum in infants,” *Aerospace Medicine*, vol. 25, no. 8, pp. 1131, 2014.

[101] X.D. Hu. “Observation of the clinical curative effect of 98 cases of children with acute bronchial pneumonia treated with Tanreqing Injection,” *Chinese Community Doctors*, vol. 30, no. 13, pp. 85, 2014.

[102] X.Q. Zhou, Z.J. Zhu. “Therapeutic effect of integrated traditional Chinese and Western medicine on acute bronchial pneumonia,” *Contemporary Medicine*, vol. 19, no. 19, pp. 105, 2013.

[103] J.H. Wei, R. Wang, J.X. Li, et al. “Treatment of 170 Cases of Bronchopneumonia with Combination of Traditional Chinese Medicine,” *Guide of China Medicine*, vol. 11, no. 12, pp. 689, 2013.

[104] L. Cai. “Therapeutic effect of Tanreqing injection on infants with bronchial pneumonia,” *For All Health*, vol. 6, no. 5, pp. 8, 2012.

[105] C. Tang. “Clinical Research of Combination Therapy of Tanreqing Injection and Antibiotics in Children with Bronchial Pneumonia and Its Effect on C-reactive Protein,” *Practical Preventive Medicine*, vol. 17, no. 9, pp. 1822, 2010.

[106] R. Chen. “Effect of Tanreqing Injection on children with bronchial pneumonia and its effect on serum CRP and TNF-α,” *China Modern Doctor*, vol. 48, no. 4, pp. 55, 2010.

[107] Y. Hu, H. Zhou, W.Q. Xu, et al. “Therapeutic effect of Tanreqing injection on infants with bronchial pneumonia,” *China Clinical Practical Medicine*, vol. 4, no. 5, pp. 192, 2010.

[108] Y. Wang, Y. Shen. “Clinical observation of Tanreqing injection combined with amoxicillin and sulbactam in the treatment of children with pneumonia,” *[Chinese and Foreign Medical Research](javascript:void(0))*, vol. 8, no. 8, pp. 92, 2010.

[109] Z.M. Zhou, L.J. Mao, Y.W. Wang. “Clinical Analysis of 56 Cases of Acute Bronchial Pneumonia in Children Treated with Tanreqing,” *Contemporary Medicine*, vol. 14, no. 8, pp. 149, 2008.

[110] H.Q. Liu. “Clinical Observation on Treatment of 96 cases of Children with Acute Bronchial Pneumonia by Tanreqing Injection,” *[Practical Journal of Cardiac Cerebral Pneumal and Vascular Disease](javascript:void(0))*, vol. 14, no. 3, pp. 225, 2006.

[111] Y.X. Liu. “Asarone Treatment of 130 Cases of Children with Bronchial Pneumonia Clinical Curative Effect Observation,” *Medical Information*, vol. 29, no. 5, pp. 271, 2016.

[112] Q.L. Zeng. “Clinical Observation on 68 Cases of Pediatric Bronchial Pneumonia Treated with Asarum,” *Yiayao Qianyan*, vol. 5, no. 27, pp. 153, 2015.

[113] Y.W. Guo. “Therapeutic effect of Xixinnao injection on children with bronchial pneumonia,” *Modern Medicine & Health*, vol. 28, no. 6, pp. 907, 2012.

[114] H.S. Piao. “Therapeutic effect of Xixinnao injection on children with asthmatic bronchial pneumonia,” *China Practical Medical*, vol. 4, no. 17, pp. 149, 2009.

[115] H. Yang, D.J. Li. “Clinical Observation of Asarone for the Treatment of Young Children Bronchopneumonla,” *[Chinese Journal of Medical Guide](javascript:void(0))*, vol. 10, no. 9, pp. 1384, 2008.

[116] S.H. Ji. “Clinical Observation on 62 Cases of Pediatric Bronchial Pneumonia Treated with Asarum,” *China New Medicine Forum*, vol. 8, no. 9, pp. 47, 2008.

[117] G.L. Wang, H. Chen, Q.S. Lu. “Therapeutic effect of α-asarone injection on children with bronchopneumonia,” *[Youjiang Medical Journal](javascript:void(0))*, vol. 35, no. 6, pp. 673, 2007.

[118] Q.J. Wang, S.Y. Song, C.X. Wang. “Therapeutic effect of α-asarone injection on asthmatic bronchopneumonia,” *[China Medical Herald](javascript:void(0))*, vol. 5, no. 18, pp. 67, 2008.

[119] B. Lin. “Therapeutic effect of Xixinnao injection on bronchial pneumonia,” *Modern Journal of Integrated Traditional Chinese and Western Medicine*, vol. 17, no. 15, pp. 2351, 2008.

[120] X.F. Yang. “Therapeutic effect of Asarum on infants with bronchial pneumonia,” *Modern Journal of Integrated Traditional Chinese and Western Medicine*, vol. 17, no. 2, pp. 221, 2008.

[121] Y.L. Wang, Y.J. Li. “Effect of Xixinnao Injection Therapy on Pneumonia in Children,” *[Journal of Applied Clinical Pediatrics](javascript:void(0))*, vol. 22, no. 10, pp. 786, 2007.

[122] X.J. Zhang. “Clinical Observation on 39 Cases of Infantile Bronchial Pneumonia Treated with Asarum Injection,” *China Modern Doctor*, vol. 45, no. 20, pp. 82, 2007.

[123] L.D. Wang, X. Wu, S. Chen. “Observation on the effect of Xiyanping in the treatment of children with bronchopneumonia,” *Chinese Journal of Rural Medicine and Pharmacy*, vol. 24, no. 10, pp. 49, 2017.

[124] L.F. Wang, W.J. Li. “Clinical efficacy of cefoperazone combined with Xiyanping in the treatment of children with bronchial pneumonia,” *[Good Health for All](javascript:void(0))*, vol. 11, no. 10, pp. 152, 2017.

[125] L.W. Pan. “Therapeutic effect and safety of Xiyanping combined with doxofylline in children with bronchopneumonia,” *[China Practical Medical](javascript:void(0))*, vol. 10, no. 26, pp. 149, 2015.

[126] S.H. Ding. “Therapeutic effect of Cefoxitin sodium for injection combined with Xiyanping in treating children bronchial pneumonia,” *Clinical Journal of Chinese Medicine*, vol. 7, no. 36, pp. 68, 2015.

[127] X. Zhao, M. Yang. “Clinical treatment effect of 89 children with bronchial pneumonia,” *World Latest Medicine Information*, vol. 15, no. 61, pp. 83, 2015.

[128] Y.Z. Chen. “Combined application of Chinese patent medicine Xiyanping and antibiotics in pneumonia,” *Yiayao Qianyan*, vol. 5, no. 35, pp. 314, 2015.

[129] W.R. Ni, H.Y. Yu. “Effect Observation on Xiyanping Combined with Second Generation Cephalosporins in Treat-ing 45 Children Cases of Bronchial Pneumonia,” *China Pharmaceuticals*, vol. 23, no. 24, pp. 105, 2014.

[130] H. Zhang. “Treatment of 70 cases of children with bronchopneumonia with Xiyanping combined with ceftriaxone sodium,” *[China Health Care & Nutrition](javascript:void(0))*, vol. 6, no. 18, pp. 3467, 2014.

[131] C.L. Luo. “Therapeutic effect of Xiyanping on children with bronchial pneumonia,” *Modern Journal of Integrated Traditional Chinese and Western Medicine*, vol. 23, no. 8, pp. 839, 2014.

[132] W.W. Li. “Therapeutic effect of Xiyanping injection on children with bronchial pneumonia,” *World Health Digest*, vol. 7, no. 11, pp. 117, 2014.

[133] F.J. Ma. “Therapeutic effect of Xiyanping injection combined with cefotiam on children with bronchial pneumonia,” *World Health Digest*, vol. 6, no. 30, pp. 187, 2013.

[134] Y. Chen. “Therapeutic effect of ceftriaxone sodium combined with Xiyanping on 60 cases of children with bronchial pneumonia,” *Guide of China Medicine*, vol. 11, no. 36, pp. 428, 2013.

[135] J.H. Zhang. “Clinical observation of Xiyanping combined with cefoperazone sulbactam in children with bronchopneumonia,” *Contemporary Medicine*, vol. 19, no. 33, pp. 137, 2013.

[136] Y.J. Lin. “Therapeutic effect of Xiyanping injection on children with bronchial pneumonia,” *Modern Medicine & Health*, vol.29, no. 9, pp. 1414, 2013.

[137] Q.J. Shi. “Treatment of 70 cases of children with bronchopneumonia with Xiyanping combined with ceftriaxone sodium,” *For All Health*, vol. 6, no. 18, pp. 27, 2012.

[138] Z.Q. Li. “Effect Observation on Xiyanping Injection in Treatmet of Bronchial Asthma,” *[Modern Diagnosis & Treatment](javascript:void(0))*, vol. 23, no. 2, pp. 87, 2012.

[139] J.C. Luo, B.J. Chen. “Therapeutic effect of Xiyanping injection on children with bronchial pneumonia,” *[Chinese Journal of Clinical Rational Drug Use](javascript:void(0))*, vol. 4, no. 34, pp. 67, 2011.

[140] G. Liu. “Therapeutic effect of Xiyanping injection on children with bronchial pneumonia,” *[Tianjin Medical Journal](javascript:void(0))*, vol. 37, no. 6, pp. 512, 2009.

[141] H. Min, J.P. Mei. “Therapeutic effect of intravenous Chuanxinlian injection on children with bronchial pneumonia,” *Shiyong Pharmacy and Clinical Remedies*, vol. 8, no. 3, pp. 46, 2005.

[142] J. Lyu. “Therapeutic effect of Xiyanping injection on children with bronchial pneumonia,” *Chinese Journal of Modern Traditional and Western Medicine*, vol. 3, no. 19, pp. 1798, 2005.

[143] D. Xu, L.P. Wang, X.H. Song, et al. “Clinical Effect of Xiyanping Injection on Bronchopneumonia in Children,” *Practical Clinical Medicine*, vol. 19, no. 2, pp. 63, 2018.

[144] X.Y. Li, Y.Q. Li, B. Ding, et al. “Effect of Xiyanping Injection combined with Azithromycin Sequential Therapy on the Serum IL-33, IL-6 and TNF-α Levels in Patients with Bronchopneumonia,” *[Progress in Modern Biomedicine](javascript:void(0))*, vol. 17, no. 4, pp. 654, 2017.

[145] C.W. Hu. “Observation on Curative Effect of Xiyanping Injection in the Treatment of Infantile Bronchial Pneumonia,” *[Chinese Medicine Modern Distance Education of China](javascript:void(0))*, vol. 14, no. 7, pp. 87, 2016.

[146] C.L. Zhu. “Therapeutic effect of Xiyanping injection on bronchial pneumonia,” *[Yiayao Qianyan](javascript:void(0))*, vol. 6, no. 28, pp. 95, 2016.

[147] J. Tian, W. Tian. “Effect Analysis of Potassium Sodium Dehydroandrographolide Succinate Combined with Piperacillin in Treatment of Children with Bronchial Pneumonia,” *World Latest Medicine Information*, vol. 18, no. 32, pp. 21, 2018.

[148] L. Long. “Clinical analysis of clinical treatment of bronchial pneumonia in children with respiratory medicine,” *Psychological Doctor*, vol. 23, no. 18, pp. 116, 2017.

[149] Y.R. Xu, S.S. Yu. “Observation of the clinical effect of Yanhuning injection in the treatment of children with bronchial pneumonia,” *Chinese Journal of Primary Medicine and Pharmacy*, vol. 23, no. 9, pp. 1401, 2016.

[150] Z.Q. Xiao, X. Liu. “Efficacy of Yanhuning as Adjuvant Treatment for Children with Bronchial Pneumonia,” *[China Foreign Medical Treatment](javascript:void(0))*, vol. 34, no. 30, pp. 134, 2015.

[151] L. Zhao. “Therapeutic effect of Yanhuning injection on children with bronchopneumonia,” *Chinese Journal of Clinical Rational Drug Use*, vol. 7, no. 14, pp. 85, 2014.

[152] Y. Liu. “A clinical analysis of treating 40 cases of children bronchial pneumonia with Yanhuning injection plus antibiotic,” *Clinical Journal of Chinese Medicine*, vol. 6, no. 8, pp. 117, 2014.

[153] L.Q. Sun. “Observation on 47 Cases of Pediatric Bronchial Pneumonia Treated with Ceftriaxone and Yanhuning,” *[Chinese Journal of Clinical Rational Drug Use](javascript:void(0))*, vol. 7, no. 2, pp. 44, 2014.

[154] F.M. Zhang. “Therapeutic effect of Yanhuning on 80 cases of children with bronchial pneumonia,” *Chinese Community Doctors*, vol. 15, no. 9, pp. 228, 2013.

[155] C.Y. Zhang. “Therapeutic effect of azithromycin combined with Yanhuning on 153 cases of children with bronchial pneumonia,” *[China Practical Medical](javascript:void(0))*, vol. 7, no. 18, pp. 187, 2012.

[156] S.Y. Pang. “Yanhuning Treats 178 Cases of Pediatric Bronchial Pneumonia,” *[China Practical Medical](javascript:void(0))*, vol. 7, no. 5, pp. 164, 2012.

[157] X. Wan. “The observation of the curative effect on the azithromycin combing with potassium sodium dehydroandroan drographolide succinate for injection treatment for children with bronchopneumonia,” *Journal of Qiqihar University of Medicine*, vol. 33, no. 2, pp. 177, 2012.

[158] F. Jiang, H.Y. Wang, L.Y. Wang. “Effect Observation of Azithromycin and Yan Hu Ning in the Treatment of Bronchial Ppneumoniain Children,” *[China Foreign Medical Treatment](javascript:void(0))*, vol. 31, no. 6, pp. 18, 2012.

[159] X.Y. Zhang, R. Zhao, G.H. Liu. “Therapeutic effect of azithromycin combined with Yanhuning on children with bronchial pneumonia,” *Chinese Journal of Misdiagnostics*, vol. 11, no. 4, pp. 807, 2011.

[160] X.L. Yu. “Clinical Analysis of Yanhuning in Treating 58 Cases of Pediatric Bronchitis,” *China Foreign Medical Treatment*, vol. 30, no. 3, pp. 115, 2011.

[161] W.J. Wang, X.H. Xu, D.M. Li, et al. “Clinical observation of Yanhuning powder injection combined with penicillin in the treatment of children with bronchial pneumonia,” *[World Health Digest](javascript:void(0))*, vol. 8, no. 7, pp. 241, 2011.

[162] Y. Pang. “Therapeutic effect of Yanhuning on 60 cases of children with bronchial pneumonia,” *China Modern Medicine*, vol. 18, no. 21, pp. 72, 2011.

[163] Y.H. Feng. “Therapeutic effect of Yanhuning injection on 48 cases of children with bronchopneumonia,” *[Chinese Journal of Modern Drug Application](javascript:void(0))*, vol. 4, no. 21, pp. 104, 2010.

[164] Y.L. Huang. “Clinical observation of Yanhuning treatment of children with bronchial pneumonia,” *Chinese Journal of Clinical Rational Drug Use*, vol. 3, no. 9, pp. 36, 2010.

[165] Y.D. Liu, Y. Wu. “Clinical Observation on 58 Cases of Infantile Bronchial Pneumonia Treated by Yanhuning Injection,” *Guide of China Medicine*, vol. 7, no. 10, pp. 2009.

[166] Z.H. Zhou. “Clinical Observation on Treatment of 35 Cases of Pediatric Bronchial Pneumonia with Yanhuning,” *[Guiding Journal of Traditional Chinese Medicine and Pharmacology](javascript:void(0))*, vol. 14, no. 5, pp. 78, 2008.

[167] Y.B. Lin. “Effect observation on Yanhuning in treatment of children's bronchopneumonia,” *International Medicine & Health Guidance News*, vol. 14, no. 7, pp. 94, 2008.

[168] B.Z. Huang, S.Q. Wei. “Therapeutic effect of Yanhuning on 68 cases of children with bronchial pneumonia,” *[Guangxi Medical Journal](javascript:void(0))*, vol. 19, no. 11, pp. 1785, 2007.

[169] Q.Y. Xiao, M. Jian, D.X. Liu. “Therapeutic effect of Yanhuning combined with cefradine on children with bronchial pneumonia,” *[Practical Clinical Journal of Integrated Traditional Chinese and Western Medicine](javascript:void(0))*, vol. 5, no. 6, pp. 30, 2005.

[170] Y.D. Zeng. “Clinical observation of Yanhuning injection for treating children with bronchial pneumonia,” *Youjiang Medical Journal*, vol. 33, no. 4, pp. 377, 2005.

[171] F.F. Zheng, K.F. Huang. “Clinical Observation on 78 Cases of Infantile Bronchial Pneumonia Treated by Yanhuning needle intravenous drip,” *International Medicine & Health Guidance News*, vol. 10, no. 18, pp. 115, 2004.

[172] J.H. Huang. “Clinical Curative Effect of Cefuroxime Combined with Yanhuning in Treat-ment of Children with Bronchial Pneumonia,” *[China Foreign Medical Treatment](javascript:void(0))*, vol. 34, no. 17, pp. 98, 2015.

[173] J.J. Gang. “Clinical observation of bronchial pneumonia in children with respiratory medicine,” *Chinese Baby*, vol. 7, no. 14, pp. 68, 2017.

[174] M. Tan. “Clinical study of azithromycin combined with Yanhuning in the treatment of children with bronchial pneumonia,” *[World Health Digest](javascript:void(0))*, vol. 25, no. 15, pp. 209, 2013.

[175] H. Hu. “Therapeutic effect of Yanhuning combined with cefradine on 40 cases of children with bronchopneumonia,” *Modern Medicine & Health*, vol. 24, no. 24, pp. 3692, 2008.

[176] Y. Tu. “Comparison of the effects of Yanhuning and Reduning on children with bronchopneumonia,” *Nei Mongol Journal of Traditional Chinese Medicine*, vol. 36, no. 17, pp. 5, 2017.

[177] Q. Lei. “To explore the efficacy of Xiyanping injection in treating children with bronchopneumonia,” *Abstract Version: Medicine and Health Care*, vol. 2, no. 3, pp. 220, 2016.

[178] A.H. Zhang. “Therapeutic effect of Xixinnao combined with Xiyanping on children with bronchial pneumonia,” *Journal of Bengbu Medical College*, vol. 40, no.1, pp. 87,2015.

[179] Y.Z. Zeng. “Therapeutic effect of Xiyanping injection on 160 cases of children with bronchial pneumonia,” *World Health Digest Medical Periodical*, vol. 7, no. 23, pp. 195, 2010.

[180] Z.Y. Yin. “Comparison of the effects of Tanreqing and Yanhuning in treating children with bronchopneumonia,” *Clinical Medicine*, vol. 30, no. 3, pp. 76, 2010.

[181] X.Q. Wang, L.H. Zhao, H. Zuo, et al. “Cost-effectiveness analysis of azithromycin and three traditional Chinese medicine preparations in the treatment of children with bronchopneumonia,” *Chinese Journal of Primary Medicine and Pharmacy*, vol. 17, no. 3, pp. 396, 2010.
